# Freedom to Stay-at-Home? Countries Higher in Relational Mobility Showed Decreased Geographic Mobility at the Onset of the COVID-19 Pandemic

**DOI:** 10.3389/fpsyg.2021.648042

**Published:** 2021-09-27

**Authors:** Jason D. Freeman, Joanna Schug

**Affiliations:** Psychological Sciences, William & Mary, Williamsburg, VA, United States

**Keywords:** relational mobility, geographic mobility, COVID-19, culture, social behavior

## Abstract

In this paper, we examine whether relational mobility (RM) (the ability for individuals to voluntarily form and terminate relationships within a given social environment) on a country level related to individuals’ tendencies to restrict their movement following the onset of the global COVID-19 pandemic and following the issuance of stay-at-home orders in their country. We use data on geographic mobility, composed of records of geolocation information provided *via* mobile phones, to examine changes in geographic mobility at the onset of the COVID-19 pandemic. We show that individuals in countries with higher RM tended to decrease their geographic mobility more than those in countries with lower RM following the onset of the COVID-19 pandemic. Similar results were found for wealth gross domestic product (GDP), but were independent of RM. These results suggest that individuals in countries with higher RM were more responsive to calls to reduce geographic mobility.

## Introduction

The onset of the COVID-19 pandemic, initiated by the rapid spread of a novel coronavirus (SARS-CoV-2) throughout the world in the early months of 2020, led to unprecedented changes in human social behavior. In the absence of a preventative vaccine or proven therapeutics during this time period, many countries attempted to curb the spread of the virus by implementing non-pharmaceutical interventions (NPIs) including broad appeals to the public to limit their geographic mobility and contact with others. However, the degree to which individuals complied with appeals to stay-at-home (SAH) varied widely across countries. Given the important role that measures such as limiting geographic mobility played in limiting the spread of COVID-19, understanding the cultural and socio-ecological factors that drive adherence to appeals to limit geographic mobility are vitally important.

In this paper, we explore whether variation in relational mobility **(RM)**, i.e., the degree to which environments provide individuals with opportunities to freely choose and exit relationships, may have impacted the extent to which individuals engaged in behavior limiting their geographic mobility at the onset of the global pandemic. Specifically, we focus on the role of the socio-ecological construct known as **RM**, defined as the number of opportunities in a given social environment for individuals to voluntarily form new relationships (Schug et al., 2009, 2010; Yuki and Schug, 2012, 2020; Oishi et al., 2015).

We examine whether RM on a country level influenced the degree to which individuals avoided engaging in activities outside of the home, such as by venturing out for retail shopping or eating out at restaurants. To do so, we use Google geolocation data derived from a sample of physical location data of all Google Maps users who have enabled Location Sharing. These data show the degree to which people in countries around the world decreased their geographic mobility after the onset of the COVID-19 pandemic, and allow us to examine whether country level RM scores generated by a prior study predict changes in geographic mobility. We note that the data presented below is correlational in nature, and as a result we cannot make definitive inferences about causation. There are also many other factors that may account for variation in geographic mobility at the onset of the COVID-19 pandemic in addition to those examined here.

Based on prior theory and research on the concept of RM, we suspected that people in societies with lower levels of RM may have less control over their social relationships, which might reduce the extent to which they are able to adhere to social distancing guidelines. In societies low in RM, individuals tend to be firmly bound to their partners in obligatory networks and social institutions characterized by systems of mutual monitoring and sanctioning (e.g., Yamagishi, 1988) and cooperative behavior is generally enforced by punishment or exclusion from the group (Yamagishi et al., 1998). As a result, behavior in these societies is often less reflective of one’s personal attitudes and preferences, as individuals are more likely to avoid any actions that may damage their relationship or reputation (e.g., Yamagishi et al., 2008, 2012). In this sense, individuals in societies with lower RM have less control over their social relationships, and their behavior will be more likely to reflect pressures from others in their environments.

Consistent with this line of thinking, individuals in societies with higher RM tend to harbor an internal locus of control and make more dispositional attributions for behavior (San Martin et al., 2019) suggesting that their behavior is driven by greater personal control and less impacted by external social influence. In this sense, higher levels of RM may afford individuals greater control over their ability to stay home. This perspective would suggest that individuals in countries with higher levels of RM may be better equipped to refrain from venturing outside of the home at the onset of the global pandemic. In summary, we sought to examine the degree to which RM on a country level may have impacted the degree to which individuals were able to reduce their geographic mobility, by decreasing their movement outside of the home once the pandemic began to worsen in their society.

## Materials and Methods

### Changes in Geographic Mobility

We used anonymized, aggregated data on a country level provided by Google’s COVID-19 Community Mobility Reports (Google LLC, 0000). Utilizing GPS data from individual smartphones, this data is presented as a daily average percent-change compared with the median value for the same day of the week during a pre-pandemic baseline (January 3rd–February 6th, 2020). Data are available beginning on February 15th, 2020. The geographic mobility data are presented as the difference in visits across six location categories on a given day compared to this pre-pandemic baseline: retail and recreation, grocery and pharmacy, parks, transit stations, workplaces, and home/residential. For example, a value of “−30” in *retail and recreation* on Sunday, March 3rd, 2020, would indicate that, on that date, visits to locations coded on Google Maps as being retail or recreation outlets were down 30% compared to the median Sunday between January 3rd and February 6th, 2020. The value for *home/residential* reflects change in the duration of time spent in a home/residence, rather than change in the number of visits to locations.

Following Chan et al. (2020), we used Principal Components Analysis with varimax rotation to compute an aggregate measure of geographic mobility from the above location categories. The overall PCA yielded a single factor with an eigenvalue of 4.46, explaining 74.3% of variance in mobility between the six categories. The extracted eigenvectors of this factor were 0.949 (*retail and recreation*), 0.870 (*grocery and pharmacy*), 0.620 (*parks*), 0.925 (*transit stations*), 0.822 (*workplaces*), and −0.941 (*home/residential*). An average across these six mobility categories, weighted by eigenvalue, was computed to create an overall geographic mobility measure each day in a particular country. Given general variability in whether SAH orders forbid excursion to parks and outdoor spaces (see Jacobsen and Jacobsen, 2020), along with the relatively low factor loading for this component, we excluded *parks* from this metric^1^.

### Relational Mobility

For RM, we used RM scores from Thomson et al.’s (2018) study, which examined participants’ perceptions of RM in 39 countries. Participants in this study were recruited *via* Facebook advertisements which invited participants to participate in a brief “quiz” about their relationships. As part of the quiz, participants responded to the RM scale, a 12-item measure that asked participants to report the availability of opportunities that other people in their immediate social environment have to voluntarily form and choose their relationships (e.g., “It is easy for them to meet new people” and “They are able to choose, according to their own preferences, the people whom they interact with in their daily life”).

### Context, Cultural, and Control Variables

Dates and strength of SAH measures at the country level were obtained from Oxford University’s Coronavirus Government Response Tracker (OxCGRT) (Hale et al., 2020). For each country on a daily basis, a value indicating the presence and strength of a SAH mandate was provided. SAH measures were tracked on an ordinal scale ranging from a value of 0 (“no measures”) to 3 (“require not leaving house with minimal exceptions”).

To control for differences in mobility patterns between weekdays and weekends, which have been previously found to be endemic to other mobile phone mobility samples (Yuan and Raubal, 2012), whether a day was a weekend or a weekday was entered into the control analysis. Each data point was dummy-coded as being on a weekend or not (0 = weekday, 1 = weekend), with the days considered “weekends” varying for majority Muslim nations (which often have a Sunday–Thursday working week), and countries with a 6-day working week such as Hong Kong (Wong and Ko, 2009).

We also include several relevant country-level controls (see Table 1), including 2019 gross domestic product (GDP per capita), as well as several controls used in Salvador et al.’s (2020) paper examining the impact of RM on the increase in COVID-19 cases on a country level. This study found that high levels of RM on a country level were associated with a greater increase in COVID-19 cases but did not examine geographic mobility. For a full listing of control variables included in our analyses see Supplementary Material.

**TABLE 1:** The impact of relational mobility on geographic mobility following the issuance of stay-at-home orders in each country.

	Model 1			Model 2		
Predictors	Estimate (SE)	95% CI	*p*	Estimate (SE)	95% CI	*p*
Intercept	32.27 (2.71)	−37.59 to −26.95	<0.001	-40.88 (19.53)	−79.16 to −2.60	0.036
Relational mobility (RM)	-1.78 (11.84)	−24.97 to 21.42	0.881	5.64 (11.46)	−16.82 to 28.09	0.623
Day from stay-at-home order	-0.42 (0.08)	−0.57 to −0.27	<0.001	-0.62 (0.08)	−0.78 to −0.46	<0.001
RM × day from stay-at-home order	-0.36 (0.14)	−0.63 to −0.08	0.011	-0.78 (0.21)	−1.20 to −0.37	<0.001
Stay-at-home (SAH) order mandatory				-19.81 (1.19)	−22.13 to −17.48	<0.001
RM × SAH order mandatory				-2.76 (6.68)	−15.85 to 10.33	0.68
Day × SAH order mandatory				0.46 (0.06)	0.33 to 0.58	<0.001
RM × day × SAH order mandatory				0.21 (0.28)	−0.33 to 0.75	0.45
Weekend				0.58 (0.51)	−0.41 to 1.57	0.254
Population density				0.30 (1.11)	−1.88 to 2.48	0.789
Population (thousands)				5.54 (3.60)	−1.51 to 12.59	0.124
Median age				0.15 (0.43)	−0.69 to 0.99	0.727
Net migration				-0.22 (0.46)	−1.12 to 0.68	0.631
GDP per capita				1.45 (3.49)	−5.39 to 8.29	0.678
% Urban population				0.13 (0.17)	−0.20 to 0.45	0.443
Random effects						
σ2	65.15			50.86		
Variance (τ00) Country	184.6			127.82		
Variance (τ00) Day from stay-at-home order	12.32			9.25		
ICC	0.75			0.73		
*N* countries	33			33		
*N* day from stay-at-home order	31			31		
Total *N*	1016			1016		
Marginal *R*^2^/conditional *R*^2^	0.060/0.766			0.337/0.821		

## Results

To examine the role of country-level RM on geographic mobility in response to the onset of the global COVID-19 pandemic, we conducted a series of linear mixed models using the package lme4 in R (Bates et al., 2015) with country and days input as random effects^2^.

### Changes in Geographic Mobility Following the Issuance of Stay-at-Home Orders

First, we examined changes in geographic mobility in response to the issuance of SAH orders by local governments. Through these analyses we sought to determine if cross-cultural differences in RM would predict responsiveness to country-level mandates aimed at reducing transmission of COVID-19 through social mixing caused by geographic mobility. We suspected that the issuance of SAH orders would be a clear and apparent cue alerting individuals of the necessity to modify their behavior.

The onset of the COVID-19 pandemic was met with great variation in how countries responded, particularly in their issuance of SAH orders. Some countries implemented SAH orders very early in the pandemic (e.g., Hong Kong), while others implemented SAH policies later (e.g., Japan) or not at all (e.g., Sweden). Thus, we sought to examine whether RM on a country level impacted changes in geographic mobility, starting on the day that SAH orders were enacted in each country.

Data included in this analysis are centered on the day that SAH orders were issued in each country. For instance, Australia first enacted a SAH order on March 24th, and Japan declared a SAH order on April 4th – both of these dates would be input as “day zero” into the model. As Sweden never initiated SAH orders, this country is omitted from the analysis. Furthermore, Hong Kong had implemented SAH orders prior to February 15th, the first date for which geographic mobility data were available. Thus, data for Hong Kong begin on day 7.

Countries varied widely in the number of days that SAH orders were in effect, and also in the number of SAH orders that were issued. For the purposes of this analysis, we sought to examine only the period of time corresponding with the first SAH order issued in each country and exclude data from any subsequent SAH period. We thus examine the first 30 days after which the first SAH order was implemented in each country.

Finally, the relative strength of SAH orders were variable both within and across countries. In some cases, it was only a recommendation that individuals SAH, whereas in other cases individuals were forbidden to leave their homes except for in special circumstances. Thus, we examined whether, on a given day, SAH orders were mandatory using data aggregated by OxCGRT, and created a dummy variable indicating whether staying at home was recommended (“recommend not leaving house”), or mandatory (“require not leaving house with exceptions for daily exercise, grocery shopping, and ‘essential’ trips”) and (“require not leaving house with minimal exceptions,” e.g., allowed to leave once a week, or only one person can leave at a time, etc.).

**FIGURE 1:**
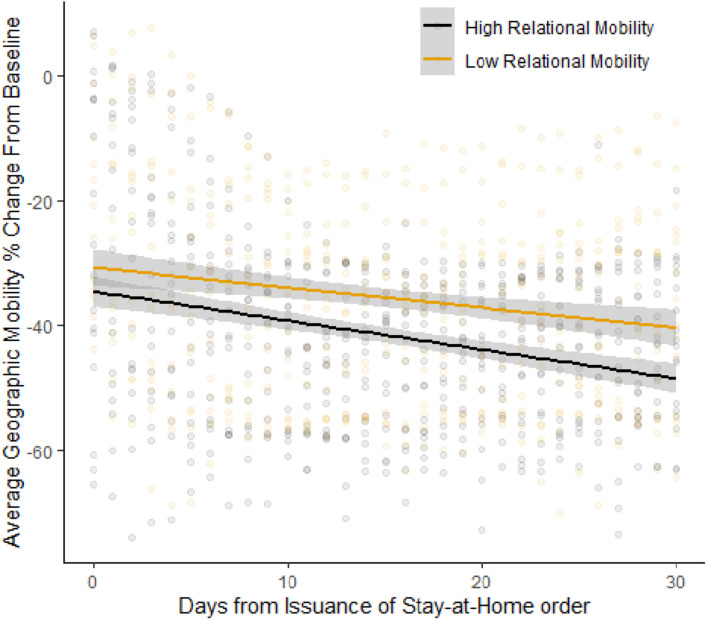
Changes in geographic mobility in the month following the issuance of stay-at-home orders in countries with high versus low levels of relational mobility.

The results are presented in Table 1. The first model shows the effect of days from SAH orders, RM, and their interaction term. The results show a significant negative effect of day (estimate = −0.42, *p* < 0.001), indicating that geographic mobility tended to decrease over time, and a significant day × RM interaction term (estimate = −0.36, *p* < 0.011), indicating that the decrease in geographic mobility following the issuance of SAH orders was greater in countries with higher RM (Figure 1). These results suggest that individuals in countries with higher RM were more likely than individuals in countries with lower RM to decrease their geographic mobility in response to the issuance of SAH orders. This result remained significant in subsequent models including parameters representing whether SAH orders were mandatory (1) or not (0) and control variables. Furthermore, there was no significant relation between RM and the strength of SAH orders, suggesting that the results cannot be explained by the tendency for countries lower in RM, to implement more stringent SAH orders.

One potential alternative explanation for this pattern is wealth. Indeed, one recent study (Oishi et al., 2021) found that residents in wealthier and more walkable neighborhoods in New York City were more likely to limit their geographic mobility at the onset of the pandemic, suggesting that more wealth may allow people greater flexibility to respond to social distancing guidelines. Given that RM on a country level is modestly correlated with GDP (Thomson et al., 2018), and that previous research has suggested that higher income is associated with more choice in one’s relationships (Bianchi and Vohs, 2016), we also examined whether country level GDP per capita accounted for the effect of RM on decreases in geographic mobility over time. The results, shown in Table 2, show that consistent with Oishi et al.’s (2021) findings, higher country GDP tended to predict greater decreases in geographic mobility over time following the issuance of SAH orders, as indicated by a significant country-level GDP per capita × day interaction (estimate = −0.13, *p* = 0.018). Importantly, the RM × day interaction term remained significant (estimate = −0.85, *p* = 0.004), indicating that the effect of RM on decreases in geographic mobility was not an artifact of cross-national variation in wealth. Country-level GDP also predicted decreases in geographic mobility at the onset of the COVID-19 pandemic, after a country had tallied 100 cases of COVID-19 (Table 3), as indicated by a GDP per capita × day interaction (estimate = −0.31, *p* < 0.001). In this analysis a RM × day interaction was also found (estimate = −0.48, *p* = 0.002), indicating that the effect of RM in this scenario is not simply the result of aggregate differences in GDP between nations.

**TABLE 2:** The impact of relational mobility and GDP per capita on geographic mobility after the issuance of stay-at-home (SAH) orders.

Predictors	Estimate (SE)	95% CI	*p*
Intercept	−23.54 (24.90)	−72.35 to 25.27	0.345
Relational mobility (RM)	26.84 (14.10)	−0.80 to 54.48	0.057
Days from stay-at-home (SAH)	−0.55 (0.09)	−0.73 to −0.38	<0.001
SAH required	−22.50 (1.96)	−26.35 to −18.66	<0.001
GDP per capita	3.26 (4.16)	−4.89 to 11.40	0.433
Weekend	0.58 (0.50)	−0.40 to 1.56	0.243
Population density	0.13 (1.30)	−2.41 to 2.67	0.92
Population	2.85 (3.91)	−4.81 to 10.50	0.466
Median age	−0.10 (0.52)	−1.13 to 0.92	0.845
Net migration	−0.19 (0.48)	−1.12 to 0.74	0.692
% Urban	0.08 (0.17)	−0.26 to 0.42	0.638
RM × days from SAH	−0.85 (0.29)	−1.42 to −0.27	0.004
RM × SAH required	−28.07 (9.50)	−46.70 to −9.45	0.003
Days from SAH × SAH required	0.42 (0.07)	0.28 to 0.57	<0.001
RM × GDP per capita (GDP)	−19.06 (14.2)	−46.89 to 8.78	0.18
Days from SAH × GDP	−0.13 (0.05)	−0.23 to −0.02	0.018
SAH required × GDP	2.13 (1.39)	−0.60 to 4.85	0.126
RM × days from SAH × SAH required	0.49 (0.35)	−0.19 to 1.16	0.161
RM × days from SAH × GDP	−0.05 (0.21)	−0.47 to 0.38	0.833
RM × SAH required × GDP	26.05 (7.83)	10.71 to 41.39	0.001
Days from SAH × SAH required × GDP	0.01 (0.07)	−0.12 to 0.14	0.845
RM × days from SAH × SAH required × GDP	−0.12 (0.31)	−0.73 to 0.49	0.703
**Random effects**			
σ2	49.43		
Variance (τ00) Country	133.43		
Variance (τ00) Days from SAH	9.62		
ICC	0.74		
*N* countries	33		
*N* days from SAH	31		
Total *N*	1016		
Marginal *R*^2^/conditional *R*^2^	0.346/0.832		

**TABLE 3:** The impact of relational mobility and GDP per capita on geographic mobility after first 100 cases of COVID-19 in a given country.

Predictors	Estimate (SE)	95% CI	*p*
Intercept	−47.71 (21.59)	−90.02 to −5.40	0.027
Relational mobility (RM)	−7.63 (11.54)	−30.24 to 14.98	0.509
Days from 100 cases	−0.26 (0.04)	−0.34 to −0.17	<0.001
SAH issued	−8.76 (0.52)	−9.78 to −7.73	<0.001
Weekend	−0.76 (0.6)	−1.94 to 0.43	0.21
Population density	0.26 (1.16)	−2.01 to 2.53	0.821
Population	6.27 (3.74)	−1.07 to 13.60	0.094
Median age	0.10 (0.45)	−0.77 to 0.97	0.822
Net migration	−0.08 (0.48)	−1.01 to 0.86	0.875
% Urban	0.23 (0.17)	−0.11 to 0.56	0.192
GDP per capita	9.68 (3.67)	2.50 to 16.87	0.008
RM × days from 100 cases	−0.48 (0.15)	−0.78 to −0.18	0.002
GDP per capita × days from 100 cases	−0.31 (0.03)	−0.37 to −0.25	<0.001
**Random effects**			
σ2	74.17		
Variance (τ00) Country	138.23		
Variance (τ00) Days from 100 cases	0.58		
ICC	0.65		
*N* countries	33		
*N* days from 100 cases	31		
Total *N*	1023		
Marginal *R*^2^/conditional *R*^2^	0.519/0.832		

### Relational Mobility and Changes in Geographic Mobility at the Onset of the COVID-19 Pandemic

Next we explored whether changes in geographic mobility would be observed during the initial onset of COVID-19 as the number of cases increased at the onset of the pandemic. We suspected that the increases in cases would correspond with greater decreases in geographic mobility, as individuals became more aware of the impact of pandemic and sought to limit their exposure to the virus outside of the home. Furthermore, we expected that people in countries with higher levels of RM (and therefore, with more control over their personal relationships) would be able to more readily decrease their geographic mobility.

To examine these possibilities, we conducted a series of linear mixed models to determine whether country-level RM predicted change in geographic mobility at the onset of the COVID-19 pandemic, following the time periods examined by Salvador et al. (2020), who examined increases in COVID-19 cases in the first 30 days after the first 100 confirmed cases of COVID-19 in each country. For this analysis, we first examined a model predicting decreases in geographic mobility with RM, day, and the RM × day interaction term to investigate whether RM on a country level was related to change over time in geographic mobility, with intercepts for country and days input as random effects. We then repeated the model including control variables, including those used in Salvador et al.’s (2020) study, along with whether each day was a weekend or not in each country and whether or not there was a SAH order in place in each country. The results of a model examining changes in geographic mobility following the first 100 cases, summarized in Table 4, showed a significant effect of day (estimate = −0.77, *p* < 0.001), indicating a decrease in geographic mobility over time, qualified by a significant interaction between day and RM (estimate = −1.27, *p* < 0.001). Inclusion of control variables in the second model did not significantly impact the results^3^.

**TABLE 4:** The impact of relational mobility on geographic mobility following the first 100 cases of COVID-19 in each country.

Predictors	Estimate (SE)	95% CI	*p*	Estimate (SE)	95% CI	*p*
Intercept	−20.6 (2.88)	−26.24 to −14.96	<0.001	−51.97 (20.28)	−91.71 to −12.23	0.01
Relational mobility (RM)	13.57 (13.71)	−13.31 to 40.44	0.322	−4.09 (11.60)	−26.83 to 18.66	0.725
Days from 100 cases	−0.77 (0.05)	−0.88 to −0.67	<0.001	−0.41 (0.04)	−0.49 to −0.34	<0.001
RM × days from 100 cases	−1.27 (0.17)	−1.60 to −0.94	<0.001	−0.70 (0.16)	−1.01 to −0.39	<0.001
Stay-at-home order in effect				−9.34 (0.55)	−10.41 to −8.27	<0.001
Weekend				−0.75 (0.63)	−1.99 to 0.50	0.239
Population density				0.24 (1.16)	−2.04 to 2.52	0.836
Population (thousands)				6.16 (3.76)	−1.20 to 13.53	0.101
Median age				0.09 (0.45)	−0.78 to 0.97	0.834
Net migration				−0.08 (0.48)	−1.02 to 0.87	0.875
GDP per capita				4.95 (3.65)	−2.21 to 12.10	0.176
% Urban population				0.23 (0.17)	−0.11 to 0.57	0.191
**Random effects**						
σ2	100.69			82.27		
Variance (τ00) Country	252.89			139.24		
Variance (τ00) Days from 100 cases	3.91			0.18		
ICC	0.72			0.63		
*N* countries	34			33		
*N* days from 100 cases	31			31		
Total *N*	1054			1023		
Marginal *R*^2^/conditional *R*^2^	0.132/0.756			0.503/0.816		

**FIGURE 2:**
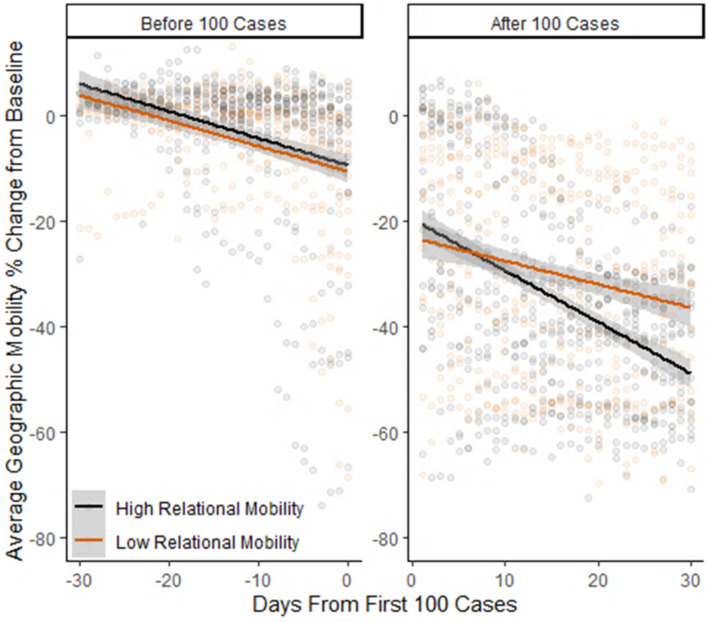
Changes in geographic mobility in 30-day periods before and after the first 100 cases, by relational mobility.

The RM × day interaction effects indicate that, as shown in the right sub-panels of Figure 2, individuals in countries with higher levels of RM showed a marked *decrease* in their geographic mobility at the onset of the COVID-19 pandemic. This decrease in geographic mobility was not observed prior to the onset of the pandemic in each country, suggesting that the tendency for people in countries higher in RM to decrease their geographic mobility was due to the onset of the pandemic rather than other factors (see Supplementary Material for additional analyses examining time periods prior to and following the onset of the COVID-19 pandemic).

These results suggest that the positive association between high RM at the country-level and increased growth rate of COVID-19 cases reported by Salvador et al. (2020) is not explained by increased geographic mobility following the onset of the COVID-19 pandemic. While their analysis showed that COVID-19 spread more rapidly in countries with higher levels of RM, our results show that high RM on a country level predicted decreases in geographic mobility, suggesting that people in countries with higher levels of RM tended to *reduce* their geographic mobility (such as by decreasing visits to restaurants and entertainment venues and staying home for longer amounts of time) at the onset of the pandemic.

### Responsiveness to Increasing COVID-19 Cases

The results of the previous analyses are consistent with the idea that people in societies with higher RM may have been better able to decrease their excursions outside of the home as the pandemic worsened in their society. That is, as awareness of the worsening pandemic increased following the issuance of SAH orders from local governments, individuals from societies with higher levels of RM may have had greater control over their social connections and were thus better able to decrease their geographic mobility in response. However, as research has suggested that evaluations of the efficacy of SAH orders are complicated by individuals voluntarily modifying their behavior before orders went into effect (Berry et al., 2021; Chin et al., 2021), we sought to examine whether the above findings would remain when examining response to the rise in cases in each region, outside of the issuance of SAH orders.

By examining the interplay between the effects of rising case levels and RM on decreases in geographic mobility, we also sought to rule out one potential alternative explanation for the findings. One possible interpretation for the decrease in geographic mobility observed in countries with higher RM may be that people in these countries were simply responding to increases in case levels [shown in Salvador et al.’s (2020) study to be greater in societies with higher RM]. If this were the case, decreases in geographic mobility in counties higher in RM may simply be an artifact resulting from the greater increase in COVID-19 cases in these regions.

To determine whether the relation between RM and geographic mobility could be explained solely by the growth in COVID-19 cases in countries with higher RM, we employed a multi-level mediation model with geographic mobility predicted by RM, as mediated by cases per 100,000 population. In this analysis RM is a level 2 variable, while cases and geographic mobility are level 1 variables. The results (shown in Supplementary Figure 1) did not find a significant indirect effect of cases per 100,000 on geographic mobility (estimate = −0.34, *p* = 0.92), which does not support the interpretation that rises in caseloads driven by higher RM drove decreases in geographic mobility.

**FIGURE 3:**
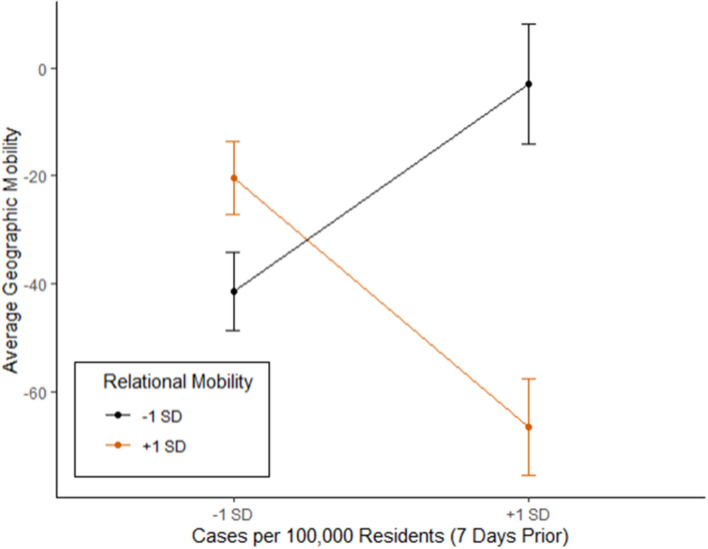
Changes in geographic mobility predicted by case levels 1 week prior, as a function of relational mobility.

We then sought to establish whether individuals from high versus low RM societies responded differently to rising case levels by reducing their geographic mobility. To do this, we conducted a series of linear mixed-effects models including country-level RM, control variables, and COVID-19 cases normalized per 100,000 population predicting change in geographic mobility. As before, analyses were limited to the first 30 days after a country reached 100 COVID-19 cases. This analysis revealed a significant interaction between cases per 100k and RM (estimate = −5.06, *p* < 0.001), such that individuals from societies higher in RM decreased their geographic mobility to a greater degree than individuals from countries lower in RM (Figure 3), particularly when case levels were high (Table 5).

**TABLE 5:** The impact of relational mobility and cases per 100,000 residents on change in geographic mobility following the first 100 cases of COVID-19 in each country.

Predictors	Estimate (SE)	95% CI	*p*	Estimate (SE)	95% CI	*p*
Intercept	−18.90 (2.97)	−24.72 to −13.08	<0.001	−49.15 (21.25)	−90.80 to −7.50	0.021
Relational mobility (RM)	17.71 (14.47)	−10.65 to 46.06	0.221	−1.92 (11.40	−24.26 to 20.42	0.866
Cases per 100,000 residents (cases)	−0.87 (0.11)	−1.09 to −0.66	<0.001	−0.60 (0.10)	−0.80 to −0.40	<0.001
Days from 100 cases (days)	−0.58 (0.04)	−0.67 to −0.50	<0.001	−0.33 (0.04)	−0.41 to −0.25	<0.001
RM × cases	−5.06 (0.97)	−6.96 to −3.16	<0.001	−4.61 (0.90)	−6.37 to −2.84	<0.001
RM × days	−0.46 (0.18)	−0.81 to −0.11	0.010	−0.01 (0.17)	−0.33 to 0.32	0.962
Cases × days	0.03 (0.00)	0.02 to 0.04	<0.001	0.02 (0.00)	0.01 to 0.03	<0.001
RM × cases × days	0.14 (0.03)	0.08 to 0.21	<0.001	0.12 (0.03)	0.06 to 0.18	<0.001
Weekend				−0.69 (0.58)	−1.83 to 0.44	0.229
Stay-at-home order in effect				−7.78 (0.52)	−8.80 to −6.77	<0.001
Population density				−0.17 (1.14)	−2.41 to 2.06	0.880
Population (thousands)				4.54 (3.68)	−2.68 to 11.76	0.218
Median age				0.33 (0.44)	−0.53 to 1.19	0.454
Net migration				−0.02 (0.47)	−0.95 to 0.90	0.960
GDP per capita				6.38 (3.58)	−0.64 to 13.40	0.075
% Urban population				0.17 (0.17)	−0.16 to 0.51	0.305
**Random effects**						
σ2	81.28			68.10		
Variance (τ00) Country	282.33			133.81		
Variance (τ00) Days from 100 cases	1.02			0.20		
ICC	0.78			0.66		
*N* countries	34			33		
*N* days from 100 cases	31			31		
Total *N*	1054			1023		
Marginal *R*^2^/conditional *R*^2^	0.223/0.827			0.550/0.848		

Next, we sought to establish temporal precedence in the observed relationship between geographic mobility and increasing case levels through a series of time lagged analyses. In these analyses, we used linear mixed effects model with countries and days input as random effects to examine whether the number of cases per capita (cases per 100,000 population) observed in each country 7 days prior^4^ would predict changes in geographic mobility (Table 6). The results of these analyses show some evidence to support the idea that people tended to decrease their activities outside of the home in response to rising case levels. Furthermore, supporting the previous findings, people in countries with high RM tended to reduce their geographic mobility in response to increased cases more so than people in countries with lower RM (estimate = −15.41, *p* < 0.001), and this difference tended to increase over time (estimate = 0.46, *p* < 0.001). These results show that individuals in countries with higher levels of RM responded to higher caseloads by decreasing their geographic mobility, more so than people in countries with lower levels of RM^5^.

**TABLE 6:** The impact of relational mobility and cases per 100,000 residents (lagged 7 days) on geographic mobility following the first 100 cases of COVID-19 in each country.

Predictors	Estimate (SE)	95% CI	*p*	Estimate (SE)	95% CI	*p*
Intercept	−20.50 (3.06)	−26.49 to −14.51	<0.001	−38.44 (21.02)	−79.64 to 2.76	0.067
Relational mobility (RM)	18.08 (14.04)	−9.44 to 45.60	0.198	−0.63 (11.28)	−22.74 to 21.48	0.955
Cases per 100k 1 week prior (prior cases)	−0.24 (0.13)	−0.50 to 0.02	0.073	−0.35 (0.12)	−0.58 to −0.11	0.005
Days from 100 cases	−0.72 (0.07)	−0.86 to −0.58	<0.001	−0.47 (0.06)	−0.58 to −0.37	<0.001
RM × prior cases	−15.41 (1.91)	−19.16 to −11.66	<0.001	−13.56 (1.86)	−17.20 to −9.92	<0.001
RM × days from 100 cases (days)	−0.44 (0.18)	−0.78 to −0.09	0.013	−0.01 (0.16)	−0.33 to 0.31	0.943
Prior cases × days	0.01 (0.00)	−0.00 to 0.02	0.052	0.01 (0.00)	0.01 to 0.02	0.001
RM × prior cases × days	0.46 (0.07)	0.33 to 0.59	<0.001	0.39 (0.06)	0.26 to 0.51	<0.001
Weekend				−0.57 (0.50)	−1.56 to 0.42	0.256
Stay-at-home order in effect				−8.18 (0.46)	−9.09 to −7.27	<0.001
Population density				0.12 (1.13)	−2.10 to 2.33	0.918
Population (thousands)				4.87 (3.65)	−2.29 to 12.03	0.183
Median age				0.08 (0.43)	−0.77 to 0.93	0.859
Net migration				−0.09 (0.47)	−1.01 to 0.82	0.845
GDP per capita				7.18 (3.55)	0.23 to 14.13	0.043
% Urban population				0.15 (0.17)	−0.18 to 0.48	0.385
**Random effects**						
σ2	85.9			72.25		
Variance (τ00) Country	266.74			131.51		
Variance (τ00) Days from 100 cases	9.99			0.65		
ICC	0.76			0.65		
*N* countries	34			33		
*N* days from 100 cases	31			31		
Total *N*	1547			1516		
Marginal *R*^2^/conditional *R*^2^	0.150/0.799			0.502/0.828		

Overall, these results support the interpretation that people in counties with higher RM responded more quickly as the pandemic worsened by reducing their geographic mobility. That is, rises in case-levels drove decreases in geographic mobility, particularly in countries with high RM, rather than the interpretation that increases in cases driven by RM caused people to decrease their geographic mobility.

## Discussion

Using Google mobility data measuring changes in geographic mobility compared to a pre-pandemic baseline, we utilized a series of linear mixed effects models to examine how country-level RM values influenced mobility after the imposition of SAH orders and following increases in cases. Our analyses showed that individuals from countries high in RM tended to decrease their geographic mobility to a greater degree at the onset of the global pandemic and following the issuance of SAH orders in their country.

The finding that people in countries with lower levels of RM were less likely to decrease their geographic mobility at the onset of the COVID-19 pandemic following the issuance of SAH orders and rises in cases suggests that social constraints in low RM societies may present an obstacle to individuals’ ability to SAH. This is consistent with the idea that behavior in countries where social relationships tend to be closed is less likely to reflect an individual’s personal desires or preferences and is more likely to reflect strategies intended to avoid negative reputation in one’s relationships (e.g., Yamagishi et al., 2008, Yamagishi et al., 2012). That is, in societies low in RM where replacement relationships are unavailable, people tend to be more sensitive to social rejection (e.g., Lou and Li, 2017), and thus behave in ways to reduce the possibility of exclusion and negative reputation (e.g., Schug et al., 2010). In this sense, just as the construct of tightness and looseness (Gelfand et al., 2011) describes the strength or weakness of cultural norms and the degree to which norms exert influence constrain an individuals’ ability to behave in accordance with their personal values and preferences, high RM might be considered to be a sort of “relational looseness” that reduces the extent to which one’s relationships exert influence over one’s behavior. In the case of this study, it is possible that increased impact of social obligations inherent in low mobility countries may have prevented individuals from staying at home at the onset of the COVID-19 pandemic, even in cases in which SAH orders were implemented.

The suggestion that higher RM may allow individuals to exert more control over their ability to limit their geographic mobility is also supported by research showing that people in low mobility countries and contexts harbor an external locus of control and tend to make more external attributions for behavior (San Martin et al., 2019). That is, individuals in countries and contexts where RM is low tend to assume that behaviors of the self and others are more likely to be determined by external forces, rather than due to factors that they can personally control. Thus, individuals who reside in high mobility contexts may be better able to exert control over their geographic mobility by adhering to SAH guidelines and otherwise reducing their excursions to entertainment venues and restaurants.

The finding that higher levels of RM may have enabled individuals to decrease their geographic mobility is similar to findings reported by Oishi et al. (2021), who show that within a large United States city, people in wealthier neighborhoods were more likely to limit their geographic mobility. Importantly, wealth on a country level is moderately associated with increased RM, and wealthier individuals in the United States have been shown to have greater control over whom they interact with, a key component of RM. We similarly find that higher GDP is associated with greater decreases in geographic mobility on a country level. Although the effect of GDP appears to be independent of the effect of RM (as reported above in Tables 3, 4), we suspect that similar forces may be at play whereby people in wealthier countries and people in countries with higher RM may have had greater ability to reduce their non-essential activities outside of the home. Of course, as this is a correlational study and there are many unmeasured variables that are not represented in these data, there are many other factors related to RM and geographic mobility that may explain why regions with higher income and RM showed reduced geographic mobility.

Superficially, the results reported in this paper may seem to contradict those reported by Salvador et al. (2020), who showed that RM predicted increased growth rates of COVID-19 at the onset of the pandemic. However, our results suggest that the decrease in geographic mobility related to RM occurred after the increase in cases observed in this previous study, and thus do not contradict those presented by Salvador et al. (2020). Our results do suggest a potential “silver lining” of RM: Although people in countries with higher RM may have been particularly vulnerable to the spread of SARS-CoV-2 at the onset of the pandemic, high RM may have allowed them to respond more nimbly as the pandemic worsened.

### Limitations and Future Research

The data presented in this manuscript should be interpreted with caution, as several limitations limit the degree to which firm conclusions may be made. First, these data are correlational in nature and, as a result, causal relationships cannot be determined. For instance, it is unclear whether events such as the issuance of SAH orders definitively caused the changes in geographic behavior reported, or whether other factors such as the degree to which information in the media increased awareness of the growing threat of COVID-19 may explain these findings. Future studies might seek to examine, for instance, how mass media coverage of the COVID-19 pandemic may have influenced behavior, by examining media content across cultures and linking the degree of coverage to changes in mobility data. Similarly, researchers might find evidence for popular discourse related to the COVID-19 pandemic by examining fluctuation in references to COVID-19, social distancing, and related terms in social media venues.

Likewise, the data on geographic mobility used in the current study also have several limitations. Importantly, the data are presented as the overall change in mobility between a given day and a pre-pandemic baseline that was the same day of the week, and as such it is not possible to examine the impact of events that may have occurred on specific days of the pre-pandemic baseline. Furthermore, the data were generated by aggregating geolocation records only for participants who used Google Maps and allowed their location to be shared with Google. While this represents a large population of data, this sample may not be inclusive of the entire population of a given country and may be subject to bias. Likewise, data on RM are also not based on a representative population, and as much may not represent the overall level of RM with perfect fidelity. In the case of this study, as the metric of RM used in the paper was compiled *via* responses from individuals on a major social network platform, and the index or geographic mobility was compiled from the behaviors of individuals who used smartphones, these populations represented in the data employed in this paper may have been younger and more technologically savvy than the general populations of their respective societies. As such, data should be interpreted with caution.

Finally, it is always possible that other underlying differences across the cultures studied, which may contribute to the differences in geographic mobility we observed in response to the onset of the COVID-19 pandemic and the imposition of SAH orders. For example, differences in RM have been shown to correlate with societal differences in general trust (Thomson et al., 2015), and there is some evidence that differences in trust contribute to differences in COVID-19 spread between countries (Elgar et al., 2020). As RM and the “openness” of social relationships in a society is also proposed to drive cultural variation in general trust (Yuki et al., 2007; Yamagishi, 2011; Thomson et al., 2018), future research may consider the potential role of trust in mediating the relationship between RM and decreases in geographic mobility. Future research should also seek to examine what other factors may further explain why countries with higher RM showed decreased geographic mobility. As described above, high RM is associated with higher internal locus of control, and lower expectations of external forces on behavior (e.g., San Martin et al., 2019), and future studies should seek to directly examine whether these factors may have impacted people’s willingness or ability to decrease their geographic mobility during the onset of the pandemic. Future research should also examine potential interplay between RM and other cultural dimensions, in particular the dimension of tightness-looseness, given evidence that more restrictive social norms prevalent in tight cultures may have been a protective factor that buffered the spread of COVID-19 (Gelfand et al., 2021).

Overall, this study found evidence that higher levels of RM, which provides individuals with more freedom over their social relationships, was associated with greater decreases in geographic mobility over time at the onset of the COVID-19 pandemic. These results illustrate the importance of examining social and cultural factors in order to help understand the factors that influenced how individuals around the world modified their behavior as a response to the pandemic. We hope that by understanding how social and cultural factors such as RM may have impacted differences in behaviors related to the pandemic will help to factors played, future generations will be better equipped to develop policies geared to limit the spread of infectious diseases.

## Data Availability Statement

Publicly available datasets were analyzed in this study. This data can be found here: https://osf.io/mbc7x/.

## Author Contributions

All authors listed have made a substantial, direct and intellectual contribution to the work, and approved it for publication.

## Conflict of Interest

The authors declare that the research was conducted in the absence of any commercial or financial relationships that could be construed as a potential conflict of interest.

## Publisher’s Note

All claims expressed in this article are solely those of the authors and do not necessarily represent those of their affiliated organizations, or those of the publisher, the editors and the reviewers. Any product that may be evaluated in this article, or claim that may be made by its manufacturer, is not guaranteed or endorsed by the publisher.

## Footnotes

1 We made the decision to exclude *parks* prior to performing data analysis. However, the results reported below do not change substantially when including *parks* in the composite mobility score.

2 We include *days* as a random effect following Salvador et al.’s (2020) paper, although we note that the overall conclusions reported below remain unchanged when *days* is input as a fixed effect.

3 The relational mobility × days interaction remained significant when the control variable representing the presence of a SAH order was excluded from the model (see Supplementary Table 3).

4 We chose a period of 7 days given evidence that the incubation period for COVID-19 found at the onset of the pandemic was approximately 7 days (Backer et al., 2020). Using a 7-day lag also allows us to control for potential day-of-the-week effects in geographic mobility.

5 We also examined an opposing model predicting current case levels with changes in geographic mobility 1 week prior, as a function of relational mobility. The results of this analysis showed no significant interaction between relational mobility and changes in geographic mobility in the previous week (estimate = −0.43, *p* = 0.142), rather, lower geographic mobility 7 days prior was associated with somewhat higher cases at present (Supplementary Table 6).
