# Supplementary material for: Freedom to Stay-at-Home? Countries Higher in Relational Mobility Showed Decreased Geographic Mobility at the Onset of the COVID-19 Pandemic
Source: Front Psychol. 2021 Sep 27;12:648042. doi: 10.3389/fpsyg.2021.648042 (PMC8502811; doi:10.3389/fpsyg.2021.648042)
Supplement: Supplementary file 1 [file Data_Sheet_1.DOCX]

***Supplementary Material***

Data and code to reproduce the analyses, and additional analyses comparing outcomes using fixed vs. random effects and for analyses using composite geographic mobility scores including the *parks* metric are available on the Open Science Framework at <https://osf.io/mbc7x/>

# Supplementary Figures and Tables

***Decrease in geographic mobility following the first death***

Models examining changes in geographic mobility following the first death in each country (Table S1) similarly show main effects of day (estimate = -0.68, *p* < .001) and a day × relational mobility interactions (estimate = -.44, *p* = .009), although the significance of this interaction was diminished when including control variables (estimate = -.16, *p* =.31) notably the presence of a SAH orders, which had a large negative impact (estimate = -9.11, *p* < .001) on geographic mobility. The following table (Table S2) shows that the interaction term remains when excluding this variable. This result suggests that Stay-at-Home orders played a strong role in diminishing geographic mobility.

***Examination time period prior to and following the onset of the COVID-19 pandemic***

To probe whether impact of relational mobility on the decrease in geographic mobility was observed at the onset of the COVID-19 pandemic, but not to decrease in geographic mobility before the onset of the pandemic, we compared periods of 30 days prior to and after the first 100 cases (see Table S3) and the first death (see Table S4), respectively. The results indicated that relational mobility predicted a significant decrease in geographic mobility following the onset of the COVID-19 pandemic, but not prior to the pandemic. For analyses examining days starting at 100 cases and 1 death, we found a significant Relational Mobility × day × onset (after 100 cases, or after 1st death) interaction term.

***GDP analysis***

As past research has shown that the ability to isolate and decrease geographic mobility is highly correlated with income (Oishi, Cha, & Schimmack, 2021), we also investigated if differences in geographic mobility between nations with differing relational mobility could be explained though differences in income across nations. In addition to analyses in our main manuscript adding an interaction term of GDP per capita across days from issuance of stay-at-home orders and the first 100 cases, we also analyzed an interaction term with the days from the first COVID-19 death (Table S5). The inclusion of this interaction term did not eliminate the effect of relational mobility after the issuance of stay-at-home orders or the first 100 cases, indicating that these effects were not the product of aggregate differences in GDP across nations studied. This analysis found a significant effect of GDP × day from 1^st^ death (estimate = -.29, *p* < .001), but not RM (estimate = .14, *p* = .352). This is likely due to the negative effect of SAH orders (estimate = -8.81, *p* < .001), similar to our previous analysis using days from 1^st^ death.

***Multi-level mediation model***

To determine whether the relation between relational mobility and geographic mobility could be explained solely by the growth in COVID-19 cases in countries with higher relational mobility, we employed a multi-level mediation model with geographic mobility predicted by relational mobility, as mediated by cases per 100,000 population. In this analysis relational mobility is a level 2 variable, while cases and geographic mobility are level 1 variables. The results (Figure S1) did not find a significant indirect effect of cases per 100,000 on geographic mobility (estimate = -.34, *p* = .92), which does not support the interpretation that the rises in cases due to relational mobility showed in a previous study (Salvador et al., 2020) drove decreases in geographic mobility in countries with higher relational mobility.

Figure S1. *Multilevel mediation model examining whether decreases in geographic mobility were driven by rising case levels in countries with higher relational mobility.*


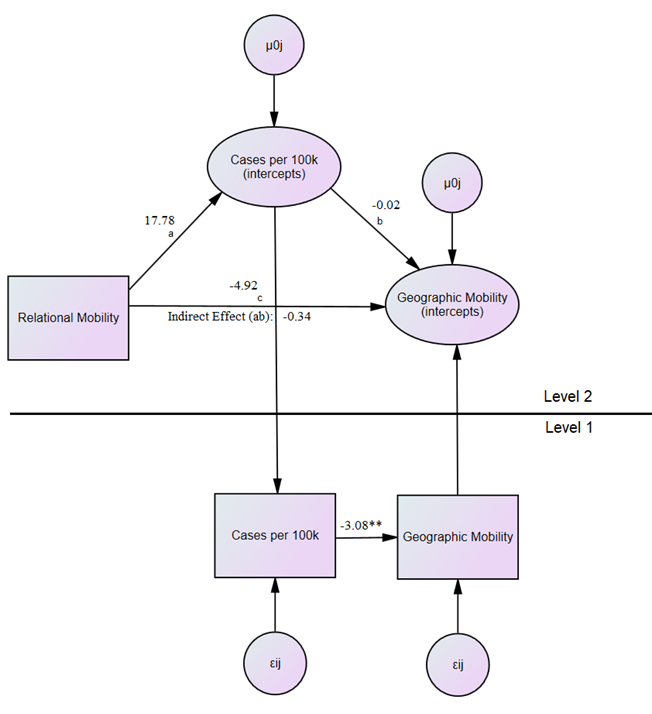


*Note.* ***p<.001*

***Lagged cases analysis***

Similar to the analysis in the main manuscript, we also examined whether changes in geographic mobility predicted changes in the number of cases per capita (per 100,000 residents) the following week (Table S6). A significant negative relationship was found between lagged geographic mobility (geographic mobility one week prior) and current cases per 100k (estimate = -.24, *p* < .001), indicating lower levels of geographic mobility were associated with *higher* cases one week later. This supports the idea decreasing geographic mobility is a function of increasing cases, but not vice versa.

**Examination of other cultural and psychological variables.**

In addition to the analysis presented in the main manuscript, we also examine the role of other variables that might impact individuals’ willingness or ability to stay home, as well as other variables that vary across cultures, including individualism and collectivism (Hofstede & Hofstede, 1984), the tightness and looseness of cultural norms (Gelfand et al., 2011). Note that because the countries for which cultural indices are available vary widely, the number of countries included in analyses for which data is available in all counties is greatly reduced, making comparisons across models difficult. We thus use interpolated values for relational mobility generated by Salvador and colleagues (2020), but these interpolated values should be evaluated with caution as they may not accurately represent actual perceptions of relational mobility in each culture.

***Analyses controlling for Tightness-Looseness***

Further analyses were conducted comparing the cultural tightness and RM as a predictor of geographic mobility after the 1st COVID-19 death in a given country (Table S7). In this analysis, tightness (estimate = -0.28, p = .048), but not RM (estimate = -0.38, *p* = .077), was found to significantly interact with date. However, it is notable that N was significantly reduced in these analyses (from 34 to 23 countries).

To mitigate the aforementioned reduction in N, the analysis above was run again using the expanded dataset with interpolated values (Table S8). Here, a significant interaction between RM (estimate = -11.19, *p* < .001) and date was found, along with an interaction between date and tightness (estimate = -0.79, *p* < .001). All effects remained when demographic controls were added in Model 2.

Similar analyses were conducted to determine if RM predicted geographic mobility similar to (or better than) cultural tightness in relation to the start of country-level outbreaks as defined by reaching 100 COVID-19 cases. As shown in Table S9, a more strongly significant interaction between RM (estimate = -0.60, *p* = 0.007) and date was found when compared to tightness and date (estimate = -0.38, p = 0.011), with these same effects remaining with the inclusion of demographic variables in Model 2. In Table S10, analyses were run again with the expanded, interpolated RM values. Here, a significant interaction between RM (estimate = -11.25, *p* <.001), and tightness (estimate = -0.69, *p* < .001), with days from 100 cases was found. These same effects remained after inclusion of demographic variables.

Analyses were run to determine if RM predicted geographic mobility similar to (or better than) cultural tightness in relation to the imposition of stay-at-home orders. As shown in Table S11, a significant interaction between tightness and date (estimate = -0.26, *p* = 0.039), but not RM and date (estimate = -0.07, p = .701), was found, with these same effects remaining with the inclusion of demographic variables in Model 2. However, as shown in Table S12, when analyses were run using the expanded dataset of interpolated RM values with a large N, a significant interaction between RM and date (estimate = -5.59, *p* <.001), as well as tightness (estimate = -0.25, *p* = .008) and date, was found. These same effects remained after inclusion of demographic variables.

***Analyses controlling for Individualism***

When comparing the influence of country-level individualism and RM in predicting changes in geographic mobility over days after the first COVID-19 death within a given country (Table S13), a significant interaction between individualism and date was found (estimate = -.01, *p* <.001), but not an interaction between RM and date (estimate = -.01, *p* = 0.955). This same effect remained when additional demographic control variables (Model 2) were added.

To account for the drop in N (from 34 to 32 countries) when including individualism, the above analyses were run again using the interpolated relational mobility scores from Salvador and colleagues expanded dataset (Table S14). Results again showed a significant interaction between individualism and date from the 1st death within a country (estimate = -0.01, *p* < .001), but not RM (estimate = 0.64, *p* = 0.345). This effect remained when additional demographic variables were included in Model 2.

As in Salvador and colleagues’ (2020) study, we also compared the ability of individualism and RM to predict changes in geographic mobility across days after the first 100 COVID-19 cases within a given country (Table S15). As shown in the table below, a significant interaction of both RM (estimate = -0.56, p < .001) and individualism (estimate = -0.01, p < .001) was found with the date from the 100th case. Both effects remained when demographic variables were included in Model 2. With the addition of interpolated values (Table S16), a significant interaction between both individualism (estimate = -.01, *p* < .001) and RM (estimate = -2.2, *p* < .001) was found with the addition of control variables.

We also tested the ability of individualism to predict changes in geographic mobility after imposition of stay-at-home orders compared with RM (Table S17). In this analysis a significant effect of individualism (estimate = -.01, p < .001) was found, but not of RM (estimate = -.22, *p* = .11) when control variables were included. When using interpolated values (Table S18), a significant effect of individualism and days from SAH order (estimate = -.01, *p* < .001), but not an effect of days from SAH x RM (estimate = -0.22, *p* = .108), was found.

A similar effect was found when analyses were conducted using an expanded dataset with interpolated RM values (Table S18). In this case, again, a significant interaction between individualism and date from SAH order (estimate = -0.01, *p* < .001) was found, but not an interaction between RM and date (estimate = -0.74, *p* = 0.222). The same interaction with individualism remained when demographic variables were added in Model 2.

References

Gelfand, M. J., Raver, J. L., Nishii, L., Leslie, L. M., Lun, J., Lim, B. C., ... & Yamaguchi, S. (2011). Differences between tight and loose cultures: A 33-nation study. *Science*, *332*(6033), 1100-1104.

Hofstede, G., & Hofstede, G. H. (1984). *Culture's consequences: International differences in work-related values* (Vol. 5). Sage.

Oishi, S., Cha, Y., & Schimmack, U. (2021). The Social Ecology of COVID-19 Cases and Deaths in New York City: The Role of Walkability, Wealth, and Race. *Social Psychological and Personality Science*. [https://doi.org/10.1177/1948550620979259](https://doi.org/10.1177%2F1948550620979259)

Salvador, C. E., Berg, M. K., Yu, Q., San Martin, A., & Kitayama, S. (2020). Relational mobility predicts faster spread of COVID-19: a 39-country study. *Psychological Science*, *31*(10), 1236-1244.

Table S1: The impact of relational mobility on geographic mobility after first death from COVID-19 in a given country

| Predictors | Estimate | (SE) | 95 % CI | p | Estimate | (SE) | 95 % CI | p |
| --- | --- | --- | --- | --- | --- | --- | --- | --- |
| (Intercept) | -20.45 | (3.12) | -26.57 – -14.33 | <.001 | -23.06 | (22.54) | -67.24 – 21.12 | .306 |
| Relational Mobility (RM) | 5.88 | (14.72) | -22.97 – 34.73 | .69 | -10.42 | (12.03) | -33.99 – 13.16 | .387 |
| Days from 1 death | -0.68 | (0.06) | -0.80 – -0.56 | <.001 | -0.34 | (0.04) | -0.42 – -0.26 | <.001 |
| RM × Days from 1 death | -0.44 | (0.17) | -0.78 – -0.11 | .009 | -0.16 | (0.16) | -0.47 – 0.15 | .31 |
| Stay-at-home issued |  |  |  |  | -9.11 | (0.54) | -10.16 – -8.05 | **<.001** |
| Weekend |  |  |  |  | -0.99 | (0.62) | -2.20 – 0.22 | .11 |
| Population Density |  |  |  |  | 0.48 | (1.21) | -1.89 – 2.85 | .691 |
| Population (Thousands) |  |  |  |  | 8.23 | (3.90) | 0.58 – 15.88 | .035 |
| Median Age |  |  |  |  | -0.04 | (0.46) | -0.95 – 0.87 | .935 |
| Net Migration |  |  |  |  | -0.20 | (0.50) | -1.18 – 0.78 | .691 |
| GDP per capita |  |  |  |  | 6.29 | (3.79) | -1.14 – 13.72 | .097 |
| % Urban Population |  |  |  |  | 0.00 | (0.18) | -0.36 – 0.35 | .987 |
| Random Effects |  |  |  |  |  |  |  |  |
| σ2 | 92.01 |  |  |  | 76.27 |  |  |  |
| τ00 Country | 292.71 |  |  |  | 150.16 |  |  |  |
| τ00 Days from 1 death | 6.35 |  |  |  | 0.67 |  |  |  |
| ICC | 0.76 |  |  |  | 0.66 |  |  |  |
| N Countries | 34 |  |  |  | 33 |  |  |  |
| N Days from 1 death | 31 |  |  |  | 31 |  |  |  |
| Total N | 1028 |  |  |  | 997 |  |  |  |
| Marginal R2 / Conditional R2 | 0.088 / 0.785 | |  |  | 0.456 / 0.817 | |  |  |

Table S2. The impact of relational mobility on geographic mobility following the COVID-19 death in each country, without controlling for Stay-at-Home Orders

| Predictors | Estimate | (SE) | 95 % CI | p |
| --- | --- | --- | --- | --- |
| (Intercept) | -2.52 | (26.55) | -54.55 – 49.51 | .924 |
| Relational Mobility (RM) | -10.26 | (14.23) | -38.14 – 17.62 | .471 |
| Days from 100 cases | -0.68 | (0.06) | -0.80 – -0.56 | <.001 |
| Weekend | -0.85 | (0.67) | -2.16 – 0.46 | .203 |
| Population density | 0.53 | (1.42) | -2.25 – 3.31 | .709 |
| Population | 10.32 | (4.50) | 1.50 – 19.15 | .022 |
| Median age | -0.26 | (0.54) | -1.32 – 0.81 | .639 |
| Net migration | -0.34 | (0.59) | -1.50 – 0.82 | .568 |
| GDP per capita | 10.52 | (4.29) | 2.11 – 18.94 | .014 |
| Urban population | -0.18 | (0.21) | -0.60 – 0.24 | .39 |
| RM × Days from 100 cases | -0.44 | (0.17) | -0.77 – 0.11 | .009 |
|  |  |  |  |  |
| Random Effects |  |  |  |  |
| σ2 | 91.96 |  |  |  |
| τ00 Country | 212.84 |  |  |  |
| τ00 Days from 100 cases | 6.35 |  |  |  |
| ICC | 0.7 |  |  |  |
| N Countries | 34 |  |  |  |
| N Days from 100 cases | 31 |  |  |  |
| Total N | 1028 |  |  |  |
| Marginal R2 / Conditional R2 | 0.327 / 0.801 | |  |  |

Table S3. The impact of relational mobility on geographic mobility prior to and after the initial 100 cases

| Predictors | Estimate | (SE) | 95 % CI | p |
| --- | --- | --- | --- | --- |
| (Intercept) | 5.73 | (2.21) | 1.40 – 10.06 | .009 |
| Relational Mobility (RM) | 16.78 | (10.41) | -3.63 – 37.19 | .107 |
| Day | -0.70 | (0.04) | -0.78 – -0.63 | <.001 |
| 100 cases | -4.12 | (0.84) | -5.77 – -2.47 | <.001 |
| RM × Day | -0.46 | (0.16) | -0.77 – -0.16 | .003 |
| RM × 100 cases | 30.44 | (3.47) | 23.64 – 37.25 | <.001 |
| Day × 100 cases | -0.06 | (0.02) | -0.11 - -0.02 | .003 |
| RM × Day × 100 cases | -0.83 | (0.09) | -1.01 – 0.65 | <.001 |
|  |  |  |  |  |
| Random Effects |  |  |  |  |
| σ2 | 141.12 |  |  |  |
| τ00 Country | 2.11 |  |  |  |
| τ00 Days from 100 cases | 120.08 |  |  |  |
| ICC | 0.46 |  |  |  |
| N Countries | 34 |  |  |  |
| N Days from 100 cases | 61 |  |  |  |
| Total N | 1871 |  |  |  |
| Marginal R2 / Conditional R2 | 0.576 / 0.773 | |  |  |

Table S4.  The impact of relational mobility on geographic mobility prior to and after the first death

| Predictors | Estimate | (SE) | 95 % CI | p |
| --- | --- | --- | --- | --- |
| (Intercept) | 5.45 | (1.58) | 2.36 – 8.54 | .001 |
| Relational Mobility (RM) | 9.09 | (6.81) | -4.26 – 22.44 | .182 |
| Day | -0.68 | (0.04) | -0.76 – -0.60 | <.001 |
| 1 Death | -3.34 | (0.97) | -5.23 – -1.44 | .001 |
| RM × Day | 0.11 | (0.15) | -0.18 – 0.40 | .455 |
| RM × 1 Death | 32.17 | (4.19) | 23.97 – 40.38 | <.001 |
| Day × 1 Death | -0.12 | (0.03) | -0.17 – -0.07 | <.001 |
| RM × Day × 1 Death | -0.97 | (0.12) | -1.21 – 0.73 | <.001 |
|  |  |  |  |  |
| Random Effects |  |  |  |  |
| σ2 | 119.07 |  |  |  |
| τ00 Country | 4.17 |  |  |  |
| τ00 Days from 1 death | 44.52 |  |  |  |
| ICC | 0.29 |  |  |  |
| N Countries | 34 |  |  |  |
| N Days from 1 death | 61 |  |  |  |
| Total N | 1791 |  |  |  |
| Marginal R2 / Conditional R2 | 0.658 / 0.757 | |  |  |

Table S5.  The impact of relational mobility and GDP per capita on geographic mobility after first death from COVID-19 in a given country

| Predictors | Estimate | (SE) | 95 % CI | p |
| --- | --- | --- | --- | --- |
| (Intercept) | -27.16 | (22.52) | -71.30 – 16.98 | .228 |
| Relational Mobility (RM) | -15.34 | (12.01) | -38.88 – 8.19 | .201 |
| Days from 1 death | -0.19 | (0.05) | -0.27 – -0.10 | <.001 |
| SAH issued | -8.81 | (0.52) | -9.82 – -7.80 | <.001 |
| Weekend | -1.02 | (0.59) | -2.18 – 0.13 | .083 |
| Population density | 0.53 | (1.21) | -1.83 – 2.90 | .658 |
| Population | 8.32 | (3.90) | 0.68 – 15.96 | .033 |
| Median age | -0.01 | (0.46) | -0.92 – 0.90 | .983 |
| Net migration | -0.20 | (0.50) | -1.18 – 0.77 | .683 |
| % Urban | 0.01 | (0.18) | -0.35 – 0.36 | .977 |
| GDP per capita | 10.56 | (3.81) | 3.08 – 18.03 | .006 |
| RM × Days from 1 death | 0.14 | (0.15) | -0.16 – 0.44 | .352 |
| GDP per capita × Days from 1 death | -0.29 | (0.03) | -0.35 – -0.23 | <.001 |
|  |  |  |  |  |
| Random Effects |  |  |  |  |
| σ2 | 69.41 |  |  |  |
| τ00 Country | 150.09 |  |  |  |
| τ00 Days from 1 death | 1.14 |  |  |  |
| ICC | 0.69 |  |  |  |
| N Countries | 33 |  |  |  |
| N Days from 1 death | 31 |  |  |  |
| Total N | 997 |  |  |  |
| Marginal R2 / Conditional R2 | 0.470 / 0.833 | |  |  |

Table S6. The impact of relational mobility and geographic mobility (lagged 7 days previous) on cases per 100,000 residents following the first 100 cases of COVID-19 in each country

| Predictors | Estimate | (SE) | 95 % CI | *p* | Estimate | (SE) | 95 % CI | *p* |
| --- | --- | --- | --- | --- | --- | --- | --- | --- |
| (Intercept) | -1.99 | (3.22) | -8.30 – 4.31 | .536 | -41.51 | (29.41) | -99.15 – 16.13 | .158 |
| Relational Mobility (RM) | -17.36 | (15.35) | -47.45 – 12.72 | .258 | -18.61 | (16.11) | -50.19 – 12.97 | .248 |
| Change in Geographic Mobility one week  prior (Prior GM) | -0.24 | (0.04) | -0.32 – -0.16 | **<.001** | -0.15 | (0.04) | -0.24 – -0.07 | **<.001** |
| Days from 100 cases (Days) | 0.57 | (0.10) | 0.37 – 0.77 | **<.001** | 0.19 | (0.11) | -0.03 – 0.41 | .090 |
| RM × Prior GM | -0.75 | (0.22) | -1.17 – -0.32 | **.001** | -0.60 | (0.22) | -1.03 – -0.18 | **.006** |
| RM × Days from 100 cases | 0.21 | (0.42) | -0.62 – 1.03 | .625 | -0.72 | (0.44) | -1.58 – 0.14 | .102 |
| Prior GM × Days | -0.00 | (0.00) | -0.01 – 0.00 | .267 | -0.01 | (0.00) | -0.01 – -0.00 | **<.001** |
| RM × Prior GM × Days | -0.03 | (0.01) | -0.05 – -0.01 | **.004** | -0.05 | (0.01) | -0.07 – -0.03 | **<.001** |
| Weekend |  |  |  |  | -9.34 | (0.55) | -10.41 – -8.27 | <.001 |
| Stay-at-Home order in effect |  |  |  |  | -0.75 | (0.63) | -1.99 – 0.50 | .239 |
| Population Density |  |  |  |  | 0.24 | (1.16) | -2.04 – 2.52 | .836 |
| Population (Thousands) |  |  |  |  | 6.16 | (3.76) | -1.20 – 13.53 | .101 |
| Median Age |  |  |  |  | 0.09 | (0.45) | -0.78 – 0.97 | .834 |
| Net Migration |  |  |  |  | -0.08 | (0.48) | -1.02 – 0.87 | .875 |
| GDP per capita |  |  |  |  | 4.95 | (3.65) | -2.21 – 12.10 | .176 |
| % Urban Population |  |  |  |  | 0.23 | (0.17) | -0.11 – 0.57 | .191 |
| Random Effects |  |  |  |  |  |  |  |  |
| σ2 | 177.12 |  |  |  | 175.19 |  |  |  |
| τ00 Country | 298.87 |  |  |  | 256.64 |  |  |  |
| τ00 Days from 100 cases | 5.04 |  |  |  | 4.26 |  |  |  |
| ICC | 0.63 |  |  |  | 0.60 |  |  |  |
| N Countries | 34 |  |  |  | 33 |  |  |  |
| N Days from 100 cases | 31 |  |  |  | 31 |  |  |  |
| Total N | 1547 |  |  |  | 1516 |  |  |  |
| Marginal R2 / Conditional R2 | 0.216 / 0.711 | |  |  | 0.337 / 0.734 | |  |  |

Table S7. The impact of relational mobility, tightness and daily COVID-19 deaths on changes in geographic mobility

| Predictors | Estimate | (SE) | 95 % CI | P | Estimate | (SE) | 95 % CI | | p |
| --- | --- | --- | --- | --- | --- | --- | --- | --- | --- |
| (Intercept) | -17.62 | (3.12) | -23.73 – -11.51 | <.001 | -40.75 | (39.68) | -118.53 – 37.02 | | .304 |
| Tightness/Looseness (TL) | 9.8 | (10.33) | -10.45 – 30.05 | .343 | 3.45 | (10.79) | -17.69 – 24.60 | | .749 |
| Days from 1 death | -0.58 | (0.05) | -0.68 – -0.47 | <.001 | -0.58 | (0.05) | -0.69 – -0.48 | | <.001 |
| Relational Mobility (RM) | 9.87 | (15.04) | -19.61 – 39.36 | .512 | 1.77 | (14.86) | -27.36 – 30.91 | | .905 |
| SAH issued | -6.99 | (0.64) | -8.25 – -5.74 | <.001 | -6.87 | (0.64) | -8.12 – -5.62 | | <.001 |
| TL × Days from 1 death | -0.28 | (0.14) | -0.56 – -0.00 | .048 | -0.29 | (0.14) | -0.57 – -0.01 | | .045 |
| RM × Days from 1 death | -0.38 | (0.22) | -0.81 – 0.04 | .077 | -0.39 | (0.22) | -0.81 – 0.04 | | .073 |
| Population density |  |  |  |  | -1.4 | (1.46) | -4.25 – 1.46 | | .339 |
| Population |  |  |  |  | 3.86 | (3.70) | -3.40 – 11.12 | | .297 |
| Median age |  |  |  |  | 0.1 | (0.66) | -1.20 – 1.40 | | .883 |
| Net migration |  |  |  |  | -0.59 | (0.95) | -2.45 – 1.26 | | .53 |
| Weekend |  |  |  |  | -1.04 | (0.75) | -2.52 – 0.44 | | .168 |
| GDP per capita |  |  |  |  | 7.89 | (4.49) | -0.90 – 16.68 | | .079 |
| % Urban Population |  |  |  |  | 0.18 | (0.30) | -0.41 – 0.77 | | .547 |
|  |  |  |  |  |  |  |  | |  |
| Random Effects |  |  |  |  |  |  |  | |  |
| σ2 | 80.48 |  |  |  | 80.34 |  |  | |  |
| τ00 Country | 161.55 |  |  |  | 105.91 |  |  | |  |
| τ00 Days from 1 death | 0.56 |  |  |  | 0.59 |  |  | |  |
| ICC | 0.67 |  |  |  | 0.57 |  |  | |  |
| N Countries | 23 |  |  |  | 23 |  |  | |  |
| N Days from 1 death | 31 |  |  |  | 31 |  |  | |  |
| Total N | 711 |  |  |  | 711 |  |  | |  |
| Marginal R2 / Conditional R2 | 0.269 / 0.758 | |  |  | 0.509 / 0.789 | | |  |  |

Table S8. The impact of interpolated relational mobility, tightness and daily COVID-19 deaths on changes in geographic mobility

| Predictors | Estimate | (SE) | 95 % CI | p | Estimate | (SE) | 95 % CI | | p | |
| --- | --- | --- | --- | --- | --- | --- | --- | --- | --- | --- |
| (Intercept) | -20.34 | (2.28) | -24.81 – -15.87 | <.001 | -38.2 | (21.58) | -80.49 – 4.09 | | .077 | |
| Tightness/Looseness (TL) | 17.43 | (6.08) | 5.52 – 29.34 | .004 | 16.25 | (7.18) | 2.19 – 30.32 | | .024 | |
| Days from 1 death | -0.53 | (0.05) | -0.64 – -0.43 | <.001 | -0.53 | (0.05) | -0.64 – -0.43 | | <0.001 | |
| Relational Mobility (RM) | 236.25 | (72.04) | 95.06 – 377.44 | .001 | 102.68 | (69.04) | -32.64 – 237.99 | | .137 | |
| SAH issued | -6.48 | (0.53) | -7.52 – -5.44 | <.001 | -6.43 | (0.53) | -7.47 – -5.39 | | <.001 | |
| TL × Days from 1 death | -0.79 | (0.09) | -0.98 – -0.61 | <.001 | -0.79 | (0.09) | -0.98 – -0.61 | | <.001 | |
| RM × Days from 1 death | -11.19 | (1.10) | -13.34 – -9.04 | <.001 | -11.21 | (1.10) | -13.36 – -9.06 | | <.001 | |
| Population density |  |  |  |  | -1.63 | (1.11) | -3.81 – 0.55 | | .143 | |
| Population |  |  |  |  | 0.60 | (0.95) | -1.25 – 2.45 | | .526 | |
| Median age |  |  |  |  | 0.47 | (0.38) | -0.28 – 1.22 | | .218 | |
| Net migration |  |  |  |  | -1.53 | (0.64) | -2.78 – -0.28 | | .016 | |
| Weekend |  |  |  |  | -0.04 | (0.61) | -1.23 – 1.16 | | .952 | |
| GDP per capita |  |  |  |  | 8.29 | (3.21) | 2.00 – 14.57 | | .01 | |
| % Urban Population |  |  |  |  | 0.01 | (0.18) | -0.33 – 0.36 | | .951 | |
|  |  |  |  |  |  |  |  | |  | |
| Random Effects |  |  |  |  |  |  |  | |  | |
| σ2 | 77.85 |  |  |  | 77.91 |  |  | |  | |
| τ00 Country | 128. |  |  |  | 78.36 |  |  | |  | |
| τ00 Days from 1 death | 3.68 |  |  |  | 3.71 |  |  | |  | |
| ICC | 0.63 |  |  |  | 0.51 |  |  | |  | |
| N Countries | 34 |  |  |  | 34 |  |  | |  | |
| N Days from 1 death | 31 |  |  |  | 31 |  |  | |  | |
| Total N | 1049 |  |  |  | 1049 |  |  | |  | |
| Marginal R2 / Conditional R2 | 0.331 / 0.752 | |  |  | 0.524 / 0.768 | | |  |  |  |

Table S9. The impact of relational mobility, Tightness and the daily COVID-19 cases on changes in geographic mobility

| Predictors | Estimate | (SE) | 95 % CI | p | Estimate | (SE) | 95 % CI | p |
| --- | --- | --- | --- | --- | --- | --- | --- | --- |
| (Intercept) | -16.29 | (2.88) | -21.94 – -10.64 | <.001 | -47.5 | (32.93) | -112.05 – 17.05 | .149 |
| Tightness/Looseness (TL) | 15.21 | (9.57) | -3.55 – 33.97 | .112 | 6.85 | (9.63) | -12.03 – 25.74 | .477 |
| Days from 1 death | -0.57 | (0.05) | -0.67 – -0.47 | <.001 | -0.58 | (0.05) | -0.68 – -0.47 | <.001 |
| Relational Mobility (RM) | 12.07 | (13.91) | -15.19 – 39.33 | .386 | 3.66 | (13.26) | -22.34 – 29.65 | .783 |
| SAH issued | -7.85 | (0.64) | -9.10 – -6.60 | <.001 | -7.68 | (0.64) | -8.93 – -6.44 | <.001 |
| TL × Days from 100 cases | -0.38 | (0.15) | -0.67 – -0.09 | .011 | -0.38 | (0.15) | -0.67 – -0.09 | .011 |
| RM × Days from 100 cases | -0.60 | (0.22) | -1.02 – -0.17 | .007 | -0.61 | (0.22) | -1.04 – -0.18 | .006 |
| Population density |  |  |  |  | -0.44 | (1.30) | -2.98 – 2.10 | .736 |
| Population |  |  |  |  | 4.32 | (3.28) | -2.12 – 10.76 | .188 |
| Median age |  |  |  |  | 0.07 | (0.59) | -1.08 – 1.23 | .902 |
| Net migration |  |  |  |  | -0.33 | (0.84) | -1.97 – 1.31 | .691 |
| Weekend |  |  |  |  | -1.02 | (0.78) | -2.55 – 0.51 | .192 |
| GDP per capita |  |  |  |  | 6.73 | (3.97) | -1.06 – 14.51 | .091 |
| % Urban Population |  |  |  |  | 0.2 | (0.26) | -0.32 – 0.72 | .451 |
|  |  |  |  |  |  |  |  |  |
| Random Effects |  |  |  |  |  |  |  |  |
| σ2 | 87.83 |  |  |  | 87.74 |  |  |  |
| τ00 Country | 136.46 |  |  |  | 82.44 |  |  |  |
| τ00 Days from 100 cases | 0.00 |  |  |  | 0.00 |  |  |  |
| ICC |  |  |  |  |  |  |  |  |
| N Countries | 23 |  |  |  | 23 |  |  |  |
| N Days from 100 cases | 31 |  |  |  | 31 |  |  |  |
| Total N | 713 |  |  |  | 713 |  |  |  |
| Marginal R2 / Conditional R2 | 0.572 / NA | |  |  | 0.710 / NA | |  |  |

Table S10. The impact of interpolated relational mobility, Tightness and the daily COVID-19 cases on changes in geographic mobility

| Predictors | Estimate | (SE) | 95 % CI | p | Estimate | (SE) | 95 % CI | | p | |
| --- | --- | --- | --- | --- | --- | --- | --- | --- | --- | --- |
| (Intercept) | -22.98 | (2.31) | -27.50 – -18.45 | <.001 | -49.91 | (19.42) | -87.97 – -11.84 | | .01 | |
| Tightness/Looseness (TL) | 16.61 | (6.41) | 4.04 – 29.17 | .01 | 15.71 | (6.90) | 2.19 – 29.23 | | .023 | |
| Days from 100 cases | -0.45 | (0.05) | -0.54 – -0.35 | <.001 | -0.45 | (0.05) | -0.54 – -0.36 | | <.001 | |
| Relational Mobility (RM) | 241.47 | (75.95) | 92.62 – 390.32 | .001 | 91.82 | (65.76) | -37.08 – 220.71 | | .163 | |
| SAH issued | -6.23 | (0.51) | -7.22 – -5.24 | <.001 | -6.12 | (0.50) | -7.11 – -5.13 | | <.001 | |
| TL × Days from 100 cases | -0.69 | (0.10) | -0.87 – -0.50 | <.001 | -0.69 | (0.10) | -0.88 – -0.50 | | <.001 | |
| RM × Days from 100 cases | -11.25 | (1.07) | -13.36 – -9.14 | <.001 | -11.28 | (1.07) | -13.39 – -9.17 | | <.001 | |
| Population density |  |  |  |  | -0.61 | (1.07) | -2.70 – 1.48 | | .568 | |
| Population |  |  |  |  | 0.41 | (0.87) | -1.30 – 2.12 | | .638 | |
| Median age |  |  |  |  | 0.62 | (0.36) | -0.08 – 1.33 | | .084 | |
| Net migration |  |  |  |  | -1.29 | (0.61) | -2.48 – -0.10 | | .033 | |
| Weekend |  |  |  |  | -0.31 | (0.58) | -1.46 – 0.83 | | .595 | |
| GDP per capita |  |  |  |  | 7.8 | (3.09) | 1.74 – 13.86 | | .012 | |
| % Urban Population |  |  |  |  | 0.07 | (0.15) | -0.23 – 0.37 | | .665 | |
|  |  |  |  |  |  |  |  | |  | |
| Random Effects |  |  |  |  |  |  |  | |  | |
| σ2 | 75.35 |  |  |  | 75.37 |  |  | |  | |
| τ00 Country | 145.30 |  |  |  | 72.94 |  |  | |  | |
| τ00 Days from 100 cases | 2.37 |  |  |  | 2.43 |  |  | |  | |
| ICC | 0.66 |  |  |  | 0.5 |  |  | |  | |
| N Countries | 36 |  |  |  | 36 |  |  | |  | |
| N Days from 100 cases | 31 |  |  |  | 31 |  |  | |  | |
| Total N | 1107 |  |  |  | 1107 |  |  | |  | |
| Marginal R2 / Conditional R2 | 0.289 / 0.760 | |  |  | 0.558 / 0.779 | | |  | |  |

Table S11. The impact of relational mobility, tightness and the imposition of stay-at-home orders on changes in geographic mobility

| Predictors | Estimate | (SE) | 95 % CI | p | Estimate | (SE) | 95 % CI | | p | |  |
| --- | --- | --- | --- | --- | --- | --- | --- | --- | --- | --- | --- |
| (Intercept) | -27.61 | (3.27) | -34.01 – -21.20 | <.001 | -38.82 | (50.13) | -137.07 – 59.44 | | .439 | |  |
| Tightness/Looseness (TL) | 4.91 | (10.06) | -14.81 – 24.63 | .626 | 2.87 | (13.49) | -23.57 – 29.32 | | .831 | |  |
| Days from Stay-at-home (SAH) | -0.54 | (0.08) | -0.69 – -0.38 | <.001 | -0.54 | (0.08) | -0.69 – -0.38 | | <.001 | |  |
| Relational Mobility (RM) | -1.26 | (14.63) | -29.93 – 27.41 | .931 | -7.75 | (18.54) | -44.08 – 28.58 | | .676 | |  |
| TL × Days from SAH | -0.26 | (0.13) | -0.52 – -0.01 | .039 | -0.27 | (0.13) | -0.52 – -0.01 | | .038 | |  |
| RM × Days from SAH | -0.07 | (0.19) | -0.44 – 0.29 | .701 | -0.07 | (0.19) | -0.44 – 0.29 | | .698 | |  |
| Population density |  |  |  |  | -1.20 | (1.84) | -4.81 – 2.41 | | .515 | |  |
| Population |  |  |  |  | 4.53 | (4.68) | -4.63 – 13.70 | | .332 | |  |
| Median age |  |  |  |  | 0.10 | (0.84) | -1.54 – 1.74 | | .904 | |  |
| Net migration |  |  |  |  | -0.19 | (1.19) | -2.53 – 2.15 | | .872 | |  |
| Weekend |  |  |  |  | 0.43 | (0.69) | -0.91 – 1.77 | | .53 | |  |
| GDP per capita |  |  |  |  | 3.82 | (5.66) | -7.28 – 14.91 | | .50 | |  |
| % Urban Population |  |  |  |  | 0.06 | (0.38) | -0.68 – 0.80 | | .875 | |  |
|  |  |  |  |  |  |  |  | |  | |  |
| Random Effects |  |  |  |  |  |  |  | |  | |  |
| σ2 | 65.33 |  |  |  | 65.39 |  |  | |  | |  |
| τ00 Country | 12.41 |  |  |  | 12.44 |  |  | |  | |  |
| τ00 Days from SAH | 154.91 |  |  |  | 171.00 |  |  | |  | |  |
| ICC | 0.72 |  |  |  | 0.74 |  |  | |  | |  |
| N Countries | 23 |  |  |  | 23 |  |  | |  | |  |
| N Days from SAH | 31 |  |  |  | 31 |  |  | |  | |  |
| Total N | 713 |  |  |  | 713 |  |  | |  | |  |
| Marginal R2 / Conditional R2 | 0.081 / 0.742 | |  |  | 0.172 / 0.783 | | |  | |  | |

Table S12. The impact of interpolated relational mobility, Tightness and the imposition of stay-at-home orders on changes in geographic mobility

| Predictors | Estimate | (SE) | 95 % CI | p | Estimate | (SE) | 95 % CI | | p | |
| --- | --- | --- | --- | --- | --- | --- | --- | --- | --- | --- |
| (Intercept) | -27.17 | (2.68) | -32.41 – -21.92 | <.001 | -53.09 | (22.84) | -97.85 – -8.33 | | .02 | |
| Tightness/Looseness (TL) | 7.23 | (7.21) | -6.91 – 21.36 | .316 | 0.60 | (8.06) | -15.19 – 16.39 | | .94 | |
| Days from Stay-at-home (SAH) | -0.47 | (0.06) | -0.59 – -0.36 | <.001 | -0.47 | (0.06) | -0.59 – -0.36 | | <.001 | |
| Relational Mobility (RM) | 51.68 | (85.42) | -115.75 – 219.10 | .545 | -100.27 | (76.56) | -250.33 – 49.78 | | .19 | |
| TL × Days from SAH | -0.25 | (0.09) | -0.43 – -0.06 | .008 | -0.25 | (0.09) | -0.44 – -0.07 | | .007 | |
| RM × Days from SAH | -5.59 | (1.03) | -7.61 – -3.57 | <.001 | -5.61 | (1.03) | -7.62 – -3.59 | | <.001 | |
| Population density |  |  |  |  | -1.53 | (1.25) | -3.98 – 0.92 | | .222 | |
| Population |  |  |  |  | 4.25 | (1.03) | 2.25 – 6.26 | | <.001 | |
| Median age |  |  |  |  | 0.68 | (0.42) | -0.16 – 1.51 | | .111 | |
| Net migration |  |  |  |  | -0.81 | (0.71) | -2.21 – 0.59 | | .255 | |
| Weekend |  |  |  |  | 0.81 | (0.57) | -0.30 – 1.92 | | .151 | |
| GDP per capita |  |  |  |  | 5.51 | (3.64) | -1.62 – 12.64 | | .13 | |
| % Urban Population |  |  |  |  | 0.01 | (0.18) | -0.35 – 0.36 | | .971 | |
|  |  |  |  |  |  |  |  | |  | |
| Random Effects |  |  |  |  |  |  |  | |  | |
| σ2 | 69.65 |  |  |  | 69.58 |  |  | |  | |
| τ00 Country | 187.31 |  |  |  | 101.99 |  |  | |  | |
| τ00 Days from SAH | 6.30 |  |  |  | 6.31 |  |  | |  | |
| ICC | 0.74 |  |  |  | 0.61 |  |  | |  | |
| N Countries | 36 |  |  |  | 36 |  |  | |  | |
| N Days from SAH | 31 |  |  |  | 31 |  |  | |  | |
| Total N | 1095 |  |  |  | 1095 |  |  | |  | |
| Marginal R2 / Conditional R2 | 0.107 / 0.764 | |  |  | 0.415 / 0.771 | | |  | |  |

Table S13. The impact of relational mobility, individualism and daily COVID-19 deaths on changes in geographic mobility

| Predictors | Estimate | (SE) | 95 % CI | p | Estimate | (SE) | 95 % CI | | p | |  |
| --- | --- | --- | --- | --- | --- | --- | --- | --- | --- | --- | --- |
| (Intercept) | -36.24 | (4.81) | -45.67 – -26.81 | <.001 | -20.88 | (23.31) | -66.58 – 24.81 | | .37 | |  |
| Individualism (IND) | 0.34 | (0.09) | 0.16 – 0.52 | <.001 | 0.23 | (0.14) | -0.04 – 0.51 | | .097 | |  |
| Days from 1 death | 0.17 | (0.07) | 0.03 – 0.30 | .018 | 0.16 | (0.07) | 0.02 – 0.30 | | .022 | |  |
| Relational Mobility (RM) | -4.14 | (11.12) | -25.93 – 17.65 | .71 | -9.24 | (11.79) | -32.35 – 13.86 | | .433 | |  |
| Stay-at-home (SAH) issued | -8.87 | (0.52) | -9.89 – -7.85 | <.001 | -8.77 | (0.52) | -9.80 – -7.75 | | <.001 | |  |
| IND × Days from SAH | -0.01 | (0.00) | -0.01 – -0.01 | <.001 | -0.01 | (0.00) | -0.01 – -0.01 | | <.001 | |  |
| RM × Days from SAH | -0.01 | (0.15) | -0.31 – 0.29 | .955 | -0.01 | (0.15) | -0.30 – 0.29 | | .966 | |  |
| Population density |  |  |  |  | 1.21 | (1.46) | -1.65 – 4.08 | | .407 | |  |
| Population |  |  |  |  | 7.29 | (3.77) | -0.09 – 14.68 | | .053 | |  |
| Median age |  |  |  |  | 0.00 | (0.45) | -0.88 – 0.87 | | .994 | |  |
| Net migration |  |  |  |  | -0.28 | (0.49) | -1.23 – 0.68 | | .571 | |  |
| Weekend |  |  |  |  | -0.98 | (0.6) | -2.15 – 0.20 | | .104 | |  |
| GDP per capita |  |  |  |  | 5.52 | (4.53) | -3.35 – 14.39 | | .222 | |  |
| % Urban Population |  |  |  |  | -0.17 | (0.19) | -0.55 – 0.21 | | .379 | |  |
|  |  |  |  |  |  |  |  | |  | |  |
| Random Effects |  |  |  |  |  |  |  | |  | |  |
| σ2 | 69.7 |  |  |  | 69.54 |  |  | |  | |  |
| τ00 Country | 155.55 |  |  |  | 137.75 |  |  | |  | |  |
| τ00 Days from 1 death | 1.21 |  |  |  | 1.25 |  |  | |  | |  |
| ICC | 0.69 |  |  |  | 0.67 |  |  | |  | |  |
| N Countries | 32 |  |  |  | 32 |  |  | |  | |  |
| N Days from 1 death | 31 |  |  |  | 31 |  |  | |  | |  |
| Total N | 966 |  |  |  | 966 |  |  | |  | |  |
| Marginal R2 / Conditional R2 | 0.355 / 0.802 | |  |  | 0.482 / 0.827 | | |  | |  | |

Table S14. The impact of interpolated relational mobility, individualism and daily COVID-19 deaths on changes in geographic mobility

| Predictors | Estimate | (SE) | 95 % CI | p | Estimate | (SE) | 95 % CI | | p | |  |
| --- | --- | --- | --- | --- | --- | --- | --- | --- | --- | --- | --- |
| (Intercept) | -36.20 | (3.65) | -43.36 – -29.04 | <.001 | -21.82 | (11.07) | -43.53 – -0.12 | | .049 | |  |
| Individualism (IND) | 0.36 | (0.08) | 0.20 – 0.52 | <.001 | 0.29 | (0.10) | 0.09 – 0.48 | | .003 | |  |
| Days from 1 death | 0.12 | (0.06) | -0.00 – 0.25 | .056 | 0.12 | (0.06) | -0.01 – 0.24 | | .062 | |  |
| Relational Mobility (RM) | -40.31 | (45.05) | -128.61 – 47.99 | .371 | -69.5 | (47.22) | -162.06 – 23.06 | | .141 | |  |
| Stay-at-home (SAH) issued | -8.80 | (0.42) | -9.62 – -7.98 | <.001 | -8.75 | (0.42) | -9.57 – -7.92 | | <.001 | |  |
| IND × Days from SAH | -0.01 | (0.00) | -0.01 – -0.01 | <.001 | -0.01 | (0.00) | -0.01 – -0.01 | | <.001 | |  |
| RM × Days from SAH | 0.64 | (0.68) | -0.69 – 1.97 | .345 | 0.63 | (0.68) | -0.70 – 1.96 | | .354 | |  |
| Population density |  |  |  |  | 1.19 | (1.04) | -0.85 – 3.23 | | .252 | |  |
| Population |  |  |  |  | 0.30 | (0.96) | -1.58 – 2.18 | | .755 | |  |
| Median age |  |  |  |  | -0.18 | (0.25) | -0.67 – 0.31 | | .475 | |  |
| Net migration |  |  |  |  | -1.14 | (0.66) | -2.44 – 0.16 | | .085 | |  |
| Weekend |  |  |  |  | -0.33 | (0.46) | -1.24 – 0.58 | | .477 | |  |
| GDP per capita |  |  |  |  | 7.23 | (2.73) | 1.87 – 12.59 | | .008 | |  |
| % Urban Population |  |  |  |  | -0.09 | (0.11) | -0.30 – 0.12 | | .414 | |  |
|  |  |  |  |  |  |  |  | |  | |  |
| Random Effects |  |  |  |  |  |  |  | |  | |  |
| σ2 | 73.86 |  |  |  | 73.86 |  |  | |  | |  |
| τ00 Country | 125.58 |  |  |  | 112.43 |  |  | |  | |  |
| τ00 Days from 1 death | 2.16 |  |  |  | 2.20 |  |  | |  | |  |
| ICC | 0.63 |  |  |  | 0.61 |  |  | |  | |  |
| N Countries | 56 |  |  |  | 56 |  |  | |  | |  |
| N Days from 1 death | 31 |  |  |  | 31 |  |  | |  | |  |
| Total N | 1707 |  |  |  | 1707 |  |  | |  | |  |
| Marginal R2 / Conditional R2 | 0.335 / 0.756 | |  |  | 0.427 / 0.775 | | |  | |  | |

Table S15. The impact of relational mobility, individualism and daily COVID-19 cases on changes in geographic mobility

| Predictors | Estimate | (SE) | 95 % CI | p | Estimate | (SE) | 95 % CI | | p | |  |
| --- | --- | --- | --- | --- | --- | --- | --- | --- | --- | --- | --- |
| (Intercept) | -35.52 | (5.04) | -45.40 – -25.64 | <.001 | -45.23 | (23.61) | -91.51 – 1.05 | | .055 | |  |
| Individualism (IND) | 0.33 | (0.10) | 0.14 – 0.52 | .001 | 0.15 | (0.14) | -0.13 – 0.43 | | .296 | |  |
| Days from 100 cases | 0.09 | (0.07) | -0.05 – 0.22 | .218 | 0.08 | (0.07) | -0.05 – 0.22 | | .237 | |  |
| Relational Mobility (RM) | -0.07 | (11.65) | -22.89 – 22.76 | .996 | -5.56 | (11.96) | -29.00 – 17.88 | | .642 | |  |
| Stay-at-home (SAH) issued | -8.27 | (0.54) | -9.33 – -7.21 | <.001 | -8.14 | (0.54) | -9.21 – -7.08 | | <.001 | |  |
| IND × Days from 100 cases | -0.01 | (0.00) | -0.01 – -0.01 | <.001 | -0.01 | (0.00) | -0.01 – -0.01 | | <.001 | |  |
| RM × Days from 100 cases | -0.56 | 0.15 | -0.86 – -0.26 | <.001 | -0.57 | (0.15) | -0.87 – -0.27 | | <.001 | |  |
| Population density |  |  |  |  | 0.29 | (1.48) | -2.62 – 3.20 | | .846 | |  |
| Population |  |  |  |  | 5.86 | (3.83) | -1.63 – 13.36 | | .125 | |  |
| Median age |  |  |  |  | 0.13 | (0.45) | -0.76 – 1.01 | | .777 | |  |
| Net migration |  |  |  |  | -0.07 | (0.49) | -1.04 – 0.90 | | .889 | |  |
| Weekend |  |  |  |  | -0.81 | (0.62) | -2.03 – 0.40 | | .189 | |  |
| GDP per capita |  |  |  |  | 5.98 | (4.59) | -3.03 – 14.98 | | .193 | |  |
| % Urban Population |  |  |  |  | 0.13 | (0.20) | -0.25 – 0.51 | | .51 | |  |
|  |  |  |  |  |  |  |  | |  | |  |
| Random Effects |  |  |  |  |  |  |  | |  | |  |
| σ2 | 75.95 |  |  |  | 75.82 |  |  | |  | |  |
| τ00 Country | 171.86 |  |  |  | 141.97 |  |  | |  | |  |
| τ00 Days from 100 cases | 0.81 |  |  |  | 0.92 |  |  | |  | |  |
| ICC | 0.69 |  |  |  | 0.65 |  |  | |  | |  |
| N Countries | 32 |  |  |  | 32 |  |  | |  | |  |
| N Days from 100 cases | 31 |  |  |  | 31 |  |  | |  | |  |
| Total N | 992 |  |  |  | 992 |  |  | |  | |  |
| Marginal R2 / Conditional R2 | 0.338 / 0.798 | |  |  | 0.494 / 0.825 | | |  | |  | |

Table S16. The impact of interpolated relational mobility, individualism and daily COVID-19 cases on changes in geographic mobility

| Predictors | Estimate | (SE) | 95 % CI | p | Estimate | (SE) | 95 % CI | | p | |  |
| --- | --- | --- | --- | --- | --- | --- | --- | --- | --- | --- | --- |
| (Intercept) | -36.65 | (3.57) | -43.64 – -29.65 | <.001 | -29.25 | (10.72) | -50.25 – -8.24 | | .006 | |  |
| Individualism (IND) | 0.35 | (0.08) | 0.19 – 0.51 | <.001 | 0.24 | (0.10) | 0.05 – 0.43 | | .013 | |  |
| Days from 100 cases | 0.11 | (0.06) | -0.01 – 0.22 | .07 | 0.11 | (0.06) | -0.01 – 0.22 | | .075 | |  |
| Relational Mobility (RM) | 8.53 | (45.34) | -80.33 – 97.38 | .851 | -37.56 | (45.81) | -127.35 – 52.23 | | .412 | |  |
| Stay-at-home (SAH) issued | -8.01 | (0.40) | -8.80 – -7.22 | <.001 | -7.93 | (0.40) | -8.72 – -7.14 | | <.001 | |  |
| IND × Days from 100 cases | -0.01 | (0.00) | -0.02 – -0.01 | <.001 | -0.01 | (0.00) | -0.02 – -0.01 | | <.001 | |  |
| RM × Days from 100 cases | -2.2 | (0.62) | -3.42 – -0.99 | <.001 | -2.20 | (0.62) | -3.42 – -0.99 | | <.001 | |  |
| Population density |  |  |  |  | 1.21 | (1.05) | -0.84 – 3.26 | | .247 | |  |
| Population |  |  |  |  | 0.03 | (0.97) | -1.87 – 1.93 | | .973 | |  |
| Median age |  |  |  |  | -0.04 | (0.24) | -0.51 – 0.44 | | .875 | |  |
| Net migration |  |  |  |  | -1.04 | (0.66) | -2.34 – 0.27 | | .119 | |  |
| Weekend |  |  |  |  | -0.54 | (0.43) | -1.38 – 0.31 | | .213 | |  |
| GDP per capita |  |  |  |  | 7.57 | (2.75) | 2.18 – 12.96 | | .006 | |  |
| % Urban Population |  |  |  |  | -0.04 | (0.11) | -0.25 – 0.17 | | .716 | |  |
|  |  |  |  |  |  |  |  | |  | |  |
| Random Effects |  |  |  |  |  |  |  | |  | |  |
| σ2 | 66.88 |  |  |  | 66.84 |  |  | |  | |  |
| τ00 Country | 135.91 |  |  |  | 115.75 |  |  | |  | |  |
| τ00 Days from 100 cases | 2.87 |  |  |  | 2.94 |  |  | |  | |  |
| ICC | 0.67 |  |  |  | 0.64 |  |  | |  | |  |
| N Countries | 59 |  |  |  | 59 |  |  | |  | |  |
| N Days from 100 cases | 31 |  |  |  | 31 |  |  | |  | |  |
| Total N | 1796 |  |  |  | 1796 |  |  | |  | |  |
| Marginal R2 / Conditional R2 | 0.315 / 0.777 | |  |  | 0.436 / 0.797 | | |  | |  | |

Table S17. The impact of relational mobility, individualism and imposition of stay-at-home orders on changes in geographic mobility

| Predictors | Estimate | (SE) | 95 % CI | p | Estimate | (SE) | 95 % CI | | p | |
| --- | --- | --- | --- | --- | --- | --- | --- | --- | --- | --- |
| (Intercept) | -42.35 | (5.11) | -52.37 – -32.34 | <.001 | -66.61 | (25.87) | -117.32 – -15.90 | | .01 | |
| Individualism (IND) | 0.24 | (0.09) | 0.06 – 0.43 | .01 | 0.16 | (0.16) | -0.15 – 0.46 | | .308 | |
| Days from SAH | 0.00 | (0.09) | -0.19 – 0.18 | .96 | -0.01 | (0.09) | -0.19 – 0.18 | | .954 | |
| Relational Mobility (RM) | -4.38 | (11.46) | -26.84 – 18.08 | .702 | -7.78 | (13.02) | -33.29 – 17.73 | | .55 | |
| IND × Days from SAH | -0.01 | (0.00) | -0.01 – -0.01 | <.001 | -0.01 | (0.00) | -0.01 – -0.01 | | <.001 | |
| RM × Days from SAH | -0.22 | (0.14) | -0.49 – 0.05 | .108 | -0.22 | (0.14) | -0.49 – 0.05 | | .11 | |
| Population density |  |  |  |  | 0.52 | (1.62) | -2.66 – 3.70 | | .748 | |
| Population |  |  |  |  | 6.52 | (4.19) | -1.68 – 14.73 | | .119 | |
| Median age |  |  |  |  | 0.51 | (0.49) | -0.46 – 1.48 | | .301 | |
| Net migration |  |  |  |  | -0.15 | (0.54) | -1.21 – 0.91 | | .78 | |
| Weekend |  |  |  |  | 0.47 | (0.56) | -0.62 – 1.56 | | .401 | |
| GDP per capita |  |  |  |  | 0.39 | (5.03) | -9.46 – 10.24 | | .938 | |
| % Urban Population |  |  |  |  | 0.12 | (0.21) | -0.30 – 0.54 | | .589 | |
|  |  |  |  |  |  |  |  | |  | |
| Random Effects |  |  |  |  |  |  |  | |  | |
| σ2 | 60.29 |  |  |  | 60.3 |  |  | |  | |
| τ00 Country | 167.77 |  |  |  | 171.13 |  |  | |  | |
| τ00 Days from SAH | 13.42 |  |  |  | 13.44 |  |  | |  | |
| ICC | 0.75 |  |  |  | 0.75 |  |  | |  | |
| N Countries | 32 |  |  |  | 32 |  |  | |  | |
| N Days from SAH | 31 |  |  |  | 31 |  |  | |  | |
| Total N | 985 |  |  |  | 985 |  |  | |  | |
| Marginal R2 / Conditional R2 | 0.315 / 0.777 | |  |  | 0.436 / 0.797 | | |  | |  |

Table S18. The impact of interpolated relational mobility, individualism and imposition of stay-at-home orders on changes in geographic mobility

| Predictors | Estimate | (SE) | 95 % CI | p | Estimate | (SE) | 95 % CI | | p | |
| --- | --- | --- | --- | --- | --- | --- | --- | --- | --- | --- |
| (Intercept) | -41.53 | (3.97) | -49.31 – -33.74 | <.001 | -46.03 | (11.62) | -68.81 – -23.25 | | <.001 | |
| Individualism (IND) | 0.30 | (0.09) | 0.12 – 0.47 | .001 | 0.22 | (0.11) | 0.01 – 0.42 | | .038 | |
| Days from SAH | -0.05 | (0.08) | -0.20 – 0.10 | .487 | -0.05 | (0.08) | -0.20 – 0.10 | | .486 | |
| Relational Mobility (RM) | -54.65 | (49.53) | -151.71 – 42.42 | .27 | -84.08 | (49.52) | -181.15 – 12.98 | | .09 | |
| IND × Days from SAH | -0.01 | (0.00) | -0.01 – -0.01 | <.001 | -0.01 | (0.00) | -0.01 – -0.01 | | <.001 | |
| RM × Days from SAH | -0.74 | (0.61) | -1.93 – 0.45 | .222 | -0.74 | (0.61) | -1.93 – 0.45 | | .225 | |
| Population density |  |  |  |  | 1.03 | (1.14) | -1.20 – 3.26 | | .365 | |
| Population |  |  |  |  | 3.39 | (1.05) | 1.32 – 5.45 | | .001 | |
| Median age |  |  |  |  | 0.34 | (0.26) | -0.18 – 0.85 | | .199 | |
| Net migration |  |  |  |  | -0.42 | (0.72) | -1.84 – 0.99 | | .559 | |
| Weekend |  |  |  |  | 0.6 | (0.42) | -0.22 – 1.42 | | .153 | |
| GDP per capita |  |  |  |  | 2.65 | (2.98) | -3.20 – 8.50 | | .374 | |
| % Urban Population |  |  |  |  | -0.07 | (0.12) | -0.30 – 0.15 | | .53 | |
|  |  |  |  |  |  |  |  | |  | |
| Random Effects |  |  |  |  |  |  |  | |  | |
| σ2 | 63.23 |  |  |  | 63.19 |  |  | |  | |
| τ00 Country | 164.14 |  |  |  | 136.93 |  |  | |  | |
| τ00 Days from SAH | 8.54 |  |  |  | 8.54 |  |  | |  | |
| ICC | 0.73 |  |  |  | 0.7 |  |  | |  | |
| N Countries | 59 |  |  |  | 59 |  |  | |  | |
| N Days from SAH | 31 |  |  |  | 31 |  |  | |  | |
| Total N | 1799 |  |  |  | 1799 |  |  | |  | |
| Marginal R2 / Conditional R2 | 0.315 / 0.777 | |  |  | 0.436 / 0.797 | | |  | |  |
